# Exploring the molecular landscape of environmental responses in the Antarctic plant *Colobanthus quitensis*: insights from metatranscriptomic analysis

**DOI:** 10.3389/fpls.2026.1774223

**Published:** 2026-03-16

**Authors:** Elisabetta Bizzarri, Silvia Proietti, Gaia Salvatore Falconieri, Carla Caruso, Laura Bertini

**Affiliations:** 1 Department of Ecological and Biological Sciences, University of Tuscia, Viterbo, Italy; 2 Institute for Sustainable Plant Protection, National Research Council of Italy, Torino, Italy

**Keywords:** *Colobanthus quitensis*, metatranscriptomics, photosynthesis, plant defence and growth, plant-associated microbiota

## Abstract

**Introduction:**

The harsh environmental conditions of Antarctica significantly influence plant responses, impacting both defence mechanisms and developmental processes. Moreover, plant-associated microbial communities further modulate physiological functions, creating a complex network of interactions. This study aimed to investigate how local environmental conditions and plant-associated microbiota shape the transcriptomic landscape of the Antarctic plant *Colobanthus quitensis*.

**Materials and methods:**

A comparative metatranscriptomic analysis was conducted on plants collected from three sites spanning a coastal-to-inland gradient of increasing climatic severity to investigate both the functional roles of differentially expressed plant genes and taxonomic profiling of the associated microbial community. In addition, the content of photosynthetic and protective pigments was quantified biochemically to assess whether environmental conditions influence the photosynthetic pathway.

**Results and discussion:**

The results suggested that Site 2 (Puchalski Station) represents a particularly favourable microenvironment that optimises the physiological performance of *C. quitensis*, supporting enhanced homeostasis and the activation of multiple stress-response strategies. Furthermore, distinct variations in microbial community composition were observed across the sites, underscoring the interplay between local environment and plant-associated microbiota.

**Conclusions:**

These findings highlight the sensitivity of *C. quitensis* to minor environmental changes and suggest that its associated microbiome could serve as an early-warning system for ecological change in Antarctica.

## 1 Introduction

Antarctica, the southernmost continent on Earth, spans around 14 million square kilometres and is almost entirely encased within the Antarctic Circle. Despite its extreme cold and harsh conditions, Antarctica supports a unique marine ecosystem, (Convey and Peck, 2019), whereas dryness and lack of soil limit the growth of plants. Terrestrial vegetation is dominated by mosses, lichens, eukaryotic algae and cyanobacteria, often thriving in areas where snow and ice melt briefly during the short summer (Green et al., 2007). Vascular plants face even greater challenges due to extremely low temperatures, strong winds, and limited sunlight. *Colobanthus quitensis* (Kunth) Bartl. and *Deschampsia antarctica* E. Desv. are the only two flowering plants endemic to Antarctica, mostly found in the milder coastal zones of Maritime Antarctica where they evolved the ability to withstand severe environmental stress without succumbing (Cavieres et al., 2016; Alberdi et al., 2002). Their southernmost populations occur on the Terra Firma Islands and the Lazarev Bay (Convey et al., 2011). These species grow primarily at suboptimal temperatures (Sáez et al., 2017) and are therefore considered Antarctic extremophiles. Studying them provides valuable insights into plant adaptation to extreme environments and expands our understanding of Antarctic terrestrial ecosystems.

Notably, plants rarely live in isolation. Rather, they form intimate and functionally significant associations with complex microbial communities that participate in mutually beneficial interactions. Within the holobiont framework, the plant host and its associated microbiota function as an integrated ecological unit shaped by co-evolutionary processes (Zilber-Rosenberg and Rosenberg, 2008; Rosenberg and Zilber-Rosenberg, 2016). Such cooperative relationships are especially crucial in resource-limited and climatically harsh environments, where they form a life-sustaining network that enhances nutrient acquisition, stress tolerance, and disease resistance, effectively extending the plant’s physiological capacity (Chauhan et al., 2023; Ali et al., 2023).

Because environmental factors, such as temperature, humidity, soil composition, wind exposure, light availability, and coastal proximity, strongly influence Antarctic plants performance, stress responses, and resilience to climate change (Ramírez et al., 2024), we have recently applied -omics approaches to investigate how distinct environmental stimuli affect the growth and functioning of *C. quitensis* meadows on King George Island (maritime Antarctica). Specifically, we first investigated the effects of increased temperature on the proteomic landscape of *C. quitensis* through an *in situ* warming experiment revealing that plants exposed to higher temperatures for one year showed improved protection against oxidative stress and enhanced photosynthetic efficiency (Bertini et al., 2021a). A subsequent comparative proteomic study on *C. quitensis* individuals from three different sites (namely Site 1, Site 2, and Site 3), distributed along a coastal-to-inland transect encompassing progressively more severe climatic conditions, revealed that local environmental variations significantly shape molecular pathways related to growth and defence (Bertini et al., 2022).

Here, we extended the latter study using a metatranscriptomic approach to assess how site-specific environmental conditions modulate plant functional activity at the gene expression level, simultaneously characterising the associated microbiota. Consistent with earlier findings (Bertini et al., 2022), Site 2 (Puchalski Station) emerged as a particularly favourable microenvironment, supporting enhanced physiological performance in *C. quitensis*. Specifically, plants at this site showed more active transcriptional profile related to growth, development, and stress mitigation. Furthermore, we highlighted that environmental factors influence both plant transcriptome reprogramming and the composition of leaf-associated microbial communities, indicating that even subtle environmental differences can substantially affect plant–microbe interactions.

## 2 Materials and methods

### 2.1 Study area and plant sampling

Sampling activity took place near the Henryk Arctowski Polish Research Station, which is located off the coast of Admiralty Bay on King George Island, Maritime Antarctica (62° 14’ S, 58° 48’ W) (**Supplementary Figure 1**). *C. quitensis* plantlets were collected from three sites (Site 1, near Arctowski Station: 62° 9’ 44.58’’ S, 58° 27’ 58.68’’ W; Site 2, near Puchalski Station: 62° 9’ 49.62’’ S, 58° 28’ 7.02’’ W; Site 3, near the Ecology Glacier: 62° 9’ 52.90’’ S, 58° 28’ 21.31’’ W) during the 2020 summer season (20 February 2020 to 20 March 2020). To minimise environmental disturbance, leaf samples were collected without removing *C. quitensis* plants from the soil. At each site, fifteen independent individuals with a diameter of 5–10 cm were randomly selected within three plots of 5 m^2^, spaced 5–8 meters apart. Leaf material from all fifteen individuals in each plot was collected using sterile forceps and pooled to form a single biological replicate. Leaves were weighed and immersed in five volumes of RNAlater^®^ solution (Sigma-Aldrich) immediately after sampling, following manufacturer’s instructions. Tissues were kept at -20 °C until their use. After RNAlater^®^ solution removal, samples were immediately immersed in liquid nitrogen, finely ground and stored at -80 °C until their use.

### 2.2 Sampling sites description

To deepen knowledge on the impact of site-specific environmental variability on *C. quitensis* growth, molecular analyses were carried out on plants collected at three different sites, namely Site 1, Site 2 and Site 3, located in King George Island, maritime Antarctica (**Supplementary Figure 1**). Site 1 is located near the Arctowski Station, in a flat, open, and waterlogged area, approximately 60 m from the shoreline and therefore subjected to strong sea-spray influence. Site 2, known as the Puchalski Station, lies about 300 m southwest of Arctowski Station, on the summit of a hill covered with moraine deposits at an altitude of approximately 50 m above sea level. Site 3 is the highest of the three sites, located at around 60 m above sea level in close proximity to the Ecology Glacier, and characterised by a glacial foreland environment with an arid microclimate. As for vegetation cover, *C. quitensis* individuals were more abundant at Site 2, while Site 1 was dominated by mosses, and Site 3 supported only sparse vegetation (**Supplementary Figure 1**). The climatic and physicochemical characteristics of the three sites are summarised in **Table 1**. Climatic data from 2018 to 2020 were mined from two Automatic Weather Stations (AWS), located at Arctowski Station (Site 1) and Puchalski Station (Site 2) (**Supplementary Table 1**; **Supplementary Figure 2**). Unfortunately, no AWS stations were in the proximity of Site 3, preventing the collection of climate data on this site. However, its higher elevation, full exposure to the prevailing south-westerly winds in the area, and proximity to the glacier make this site more inhospitable for plant colonisation.

**Table 1:** Overview of the climate and soil properties of the three sampling sites.

Parameters	Site 1	Site 2	Site 3
Mean Air Temperature (°C)	-0.95	-1.62	N/A
Mean Max Air T (°C)	1.43	0.85	N/A
Mean Min Air T (°C)	-3.19	-3.78	N/A
Mean humidity (%)	81.55	79.59	N/A
Wind (m/s)	5.11	5.76	N/A
Distance from the Coastline (m)	60 m	300 m	550 m
Altitude (masl)	0 m	50 m	60 m
Soil Textural Class	Sandy loam	Loamy sand	Sandy loam
Soil pH	4.77	5.37	6.11
Soil Classification	Very strongly acidic	Strongly acidic	Slightly acidic

### 2.3 Determination of chlorophylls and carotenoids content

Determination of chlorophylls and carotenoids was conducted on leaf samples, according to the protocol of Porra et al. (1989). Briefly, 0.15 g of leaves were homogenised in liquid nitrogen and then 1.5 mL of 80% acetone was added. The homogenate was centrifuged at 7000 rpm for 3 min, supernatant was then recovered while the pellet was resuspended again in 80% acetone (0.5 to 1 mL). The acetone resuspension and centrifugation steps were repeated until the pellets were fully decolorised to extract all the pigments they contained. The final supernatant volume of each sample was measured and used to calculate concentration of chlorophylls and carotenoids in the starting plant material as previously described (Falconieri et al., 2024).

### 2.4 Determination of anthocyanins content

Anthocyanins content was determined in leaf samples following the procedure of with some modifications. Briefly, 200 mg of fresh leaf tissues were cold homogenised and resuspended in 2 ml of 0.1% HCl in methanol. At the end of the extraction, the suspension was collected in Eppendorf tube and centrifuged at 10000 rpm for 10 min; 100 μL of supernatant were added to 900 μL of methanol acidified as above and the absorbance at 536 nm was divided by the molar extinction coefficient of cyanidin-3-glucoside in the same solvent (34300 mol^-1^ cm^-1^) to quantify anthocyanin content.

### 2.5 Statistical analysis

One-way ANOVA followed by Tukey’s multiple comparison test was performed using GraphPad Prism 9.5 (GraphPad Software Inc., San Diego, CA, USA) to determine the statistical significance of differences in chlorophylls, carotenoids and anthocyanins content between the three sites. Three technical replicates were performed for each of three biological replicates.

### 2.6 Metatransciptomic analysis of *C. quitensis* leaves

Total RNA was extracted from leaf samples using the Nucleospin^®^ RNAPlant kit (Macherey–Nagel, Düren, Germany), starting from 100 mg of finely ground leaf samples, in accordance with the manufacturer’s instructions. Extracted RNA was treated with DNase from the same kit to remove genomic DNA contamination. RNA concentration and quality were assessed on Nanodrop NanoReady Touch FC-3100 (Hangzhou LifeReal Biotechnology Co., Hangzhou, Zhejiang, China), its integrity was verified by agarose gel electrophoresis, whereas the absence of DNA contamination was assessed using 100 ng of total RNA as a template in a PCR reaction using *EF1a* gene-specific primers for amplification (Forward primer: GGCCTAATCACACCGGTTTC; Reverse primer: CTGGTTTTGAGGGTGACAACA) (Bertini et al., 2021b). RNA sequencing was carried out using the Illumina TruSeq Stranded mRNA kit, in conjunction with the QIAseq FastSelect - rRNA Plant Kit (Qiagen, Hilden, Germany) to eliminate ribosomal sequences. The sequencing process employed the Illumina S1 100PE Novaseq flowcell (BMR Genomics, Padua, Italy). Reads preprocessing was performed using fastp v0.20.0 (Chen et al., 2018) to remove residual adapter sequences and filter low-quality data. We applied specific parameters to ensure high-quality output: a phred quality score threshold of 20, an unqualified base limit of 30%, and a required average quality of 25. Additionally, a low-complexity filter was applied (complexity_threshold=30). After filtering, the mean percentage of retained high-quality reads was 96.5%. Sequencing data were submitted to the Sequence Read Archive (SRA) database (https://www.ncbi.nlm.nih.gov/sra/) of NCBI (https://www.ncbi.nlm.nih.gov/) under the accession number PRJNA1230071. The processed reads that passed the filtering step were then mapped to the *C. quitensis* Transcriptome Shotgun Assembly (TSA) (GenBank: GCIB00000000.1) (Arthofer et al., 2015) using Salmon v1.4.0 (Patro et al., 2017) with standard parameters, except for enabling the validate Mappings option to enhance alignment sensitivity. RNAs differential analysis (pairwise comparisons) was performed using edgeR (McCarthy et al., 2012) with raw counts in each comparison. Transcripts with pvalue ≤ 0.001, FDR ≤ 0.1 and a threshold of log2 (Fold Change) > |1| were considered as differentially expressed. To discern differentially expressed genes (DEGs) belonging to the plant kingdom among all DEGs, an annotation process was carried out using the NCBI genomic database, which includes four kingdoms (virus, bacteria, fungi, and plants). The annotation was performed using the Diamond tool (Buchfink et al., 2021), generating a gtf file with genes predictions and annotations. To conduct a robust gene enrichment analysis on plant DEGs, we used a reference set of *Arabidopsis thaliana* putative orthologous genes relative to our DEGs. To achieve this, we adopted the widely used diamond blast reciprocal best hit strategy (Wall et al., 2003; Ward and Moreno-Hagelsieb, 2014; Hernández-Salmerón and Moreno-Hagelsieb, 2020). The alignment was performed against the *Arabidopsis* proteome available on The *Arabidopsis* Information Resource (TAIR) website, using the last available annotation set, Araport11 (Cheng et al., 2017). The functions and metabolic pathways associated with DEGs were explored through ShinyGO 0.81 software (Ge et al., 2020) using *Arabidopsis* as the reference species for each paired comparison. Through this analysis, we identified KEGG (Kyoto Encyclopedia of Genes and Genomes) pathways (Kanehisa and Goto, 2000) and GO terms (Harris et al., 2004) significantly enriched in all analysed samples.

### 2.7 Analysis of bacterial community associated to *C. quitensis* leaves

To retrieve bacterial sequences from the metatranscriptomic dataset, an additional filtering phase, consisting of three sequential steps, was carried out to improve data quality and remove potential contaminants. Briefly, data were firstly processed using the fastp software (v. 0.23.4) to remove low-quality reads. Subsequently, the reads were aligned using bowtie2 (v.2.2.3) against the Silva database (v.138) (Quast et al., 2013; Yilmaz et al., 2014) to remove ribosomal material and then against the human genome (v.105 Ensembl) to eliminate external contaminants from the samples. The resulting data were provided to Kraken2 (v 2.1.3) (Wood et al., 2019) for taxonomic classification, with the confidence parameter set to 0.05, while leaving the remaining parameters at default settings. The results obtained were imported into the R environment for diversity and differential abundance analyses. Diversity metrics were generated using a combination of the “phyloseq” and “mia” libraries for both alpha and beta diversity indices. Differential abundance analysis was carried out using three complementary statistical approaches: DESeq2 (Love et al., 2014), AncomBC (Law et al., 2014), and MaAsLin2 (Mallick et al., 2021). Results from the three methods were filtered for p-value < 0.05 and compared to derive an overall consensus score. A maximum score of 3 was assigned to the differentially abundant (DA) taxa identified by all three methods; DA taxa with a score of 2 were also considered (**Supplementary Table 2**). Lastly, heatmaps were generated using hierarchical clustering to classify samples based on abundance data. For this phase, DA taxa with a score of 3 and the highest significance values were selected.

## 3 Results and discussion

### 3.1 Photosynthetic pigments profile

Photosynthetic pigments, including chlorophylls and accessory pigments such as carotenoids, xanthophylls, and phycobilins, play a key biological role in photosynthesis by expanding the usable light spectrum and contributing to photoprotection, while their composition varies in response to environmental factors such as temperature, UVB radiation, and nutrient and water availability (Simkin et al., 2022).

**Figure 1:**
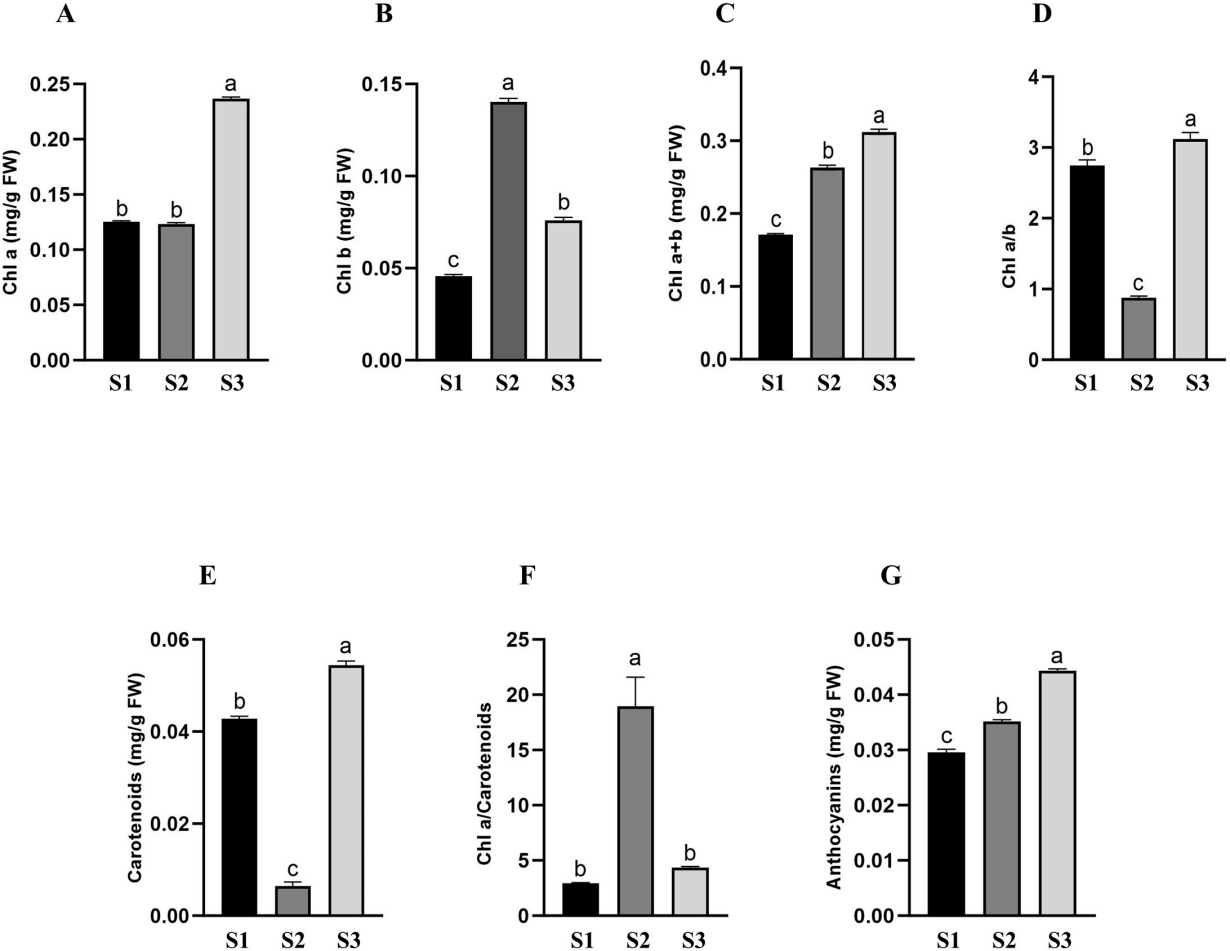
Photosynthetic pigment content in plants harvested in site 1, site 2 and site 3. **(A)** chlorophyll a (Chl a) content; **(B)** chlorophyll b (Chl b) content; **(C)** total chlorophyll content; **(D)** chlorophyll a/b ratio; **(E)** Carotenoids content; **(F)** chlorophyll a/carotenoid ratio; **(G)** Anthocyanins content. Letters above the bars indicate significant differences between sites (One-way analysis of variance, Tukey’s test; n=3; p value <0,0001).

To highlight whether the natural environment of *C. quitensis* growing area affects its photosynthetic pigments composition, we determined the amount of Chl a and Chl b as well as that of carotenoids and anthocyanins (**Figure 1**). Specifically, the quantity of Chl a is significantly higher in plants from Site 3 compared to the other two sites (**Figure 1A**), while the quantity of Chl b is higher in Site 2 compared to the other two (**Figure 1B**). As for the total chlorophyll content, there is a progressive increase in these pigments from Site 1 to Site 3 (**Figure 1C**). It is well acknowledged that chlorophyll concentration (Chl a, Chl b, and Chl a+b) can be used as a marker to evaluate the efficiency of light use by plants and, consequently, their productivity. In particular, high levels of Chl b in chloroplasts enable organisms to transform a broader spectrum of solar energy into chemical energy and this is thought to be an adaptation to shade (Kitajima and Hogan, 2003). Furthermore, since Chl a and Chl b are the main photosynthetic pigments of the reaction centres (RCs) and the light-harvesting complexes (LHCs), respectively, the Chl a/b ratio largely indicates how plants allocate their resources among various protein complexes in the photosystem (PS), as well as the functional balance between light harvesting and electron transport (Kume et al., 2018). As a consequence, plants are able to adjust their LHC or RC allocation in order to respond to the changing environmental conditions. In our work, we highlighted a Chl a/b ratio of 0.7 in Site 2 plants, while higher ratios were disclosed in both Site 1 and Site 3 plants (2.75 and 3.12, respectively) (**Figure 1D**). It is widely recognised that plants generally maintain a low chlorophyll a/b ratio, since RCs are more expensive compared to LHCs (Kitajima and Hogan, 2003; Larkum et al., 2018). In addition, it has been reported that species belonging to the Compositae family, predominantly found in cold Alpine regions, show a low chlorophyll a/b ratio which may help them better cope with potential damage resulting from low temperatures and intense radiation while maintaining their productivity (Castrillo, 2006). In addition, the leaf concentration (Chl a, Chl b, and Chl a+b) and composition (Chl a/b ratio) of chlorophylls can be influenced by numerous environmental factors, such as light, water availability, and temperature (Croft et al., 2017). Based on our results, we can hypothesise that the higher concentration of Chl b in Site 2 plants, and therefore a lower Chl a/b ratio, may reflect a reallocation of pigments in favour of a less costly process, leading to greater overall photosynthetic efficiency.

Alongside chlorophylls, carotenoids (Car) are essential plant pigments that play a dual role within the photosynthetic machinery of plants, being involved in both light harvesting and photoprotection from damage caused by light excess and oxidative harm (Sun et al., 2022). They also serve multiple roles in non-photosynthetic plant parts, functioning as protectors against light damage, antioxidants, agents that attract pollinators with their colour, and as the building blocks for various plant hormones (Gómez-Sagasti et al., 2023). As shown in **Figures 1E**, Site 2 plants exhibit a very low carotenoid content compared to the other samples, suggesting that they may not need to produce high levels of photoprotective pigments, as they likely experience less photooxidative stress. Besides the Chl a/Chl b ratio, also the Chl a/Car ratio is an important indicator of the plant’s physiological state and their ability to cope with environmental conditions. Indeed, as highlighted in literature, the Chl a/Car ratio undergoes a decrease under stress (Zhou et al., 2019; Richardson et al., 2002). In our experimental conditions, the Chl a/Car ratio of Site 2 plants is higher than in other samples (**Figure 1F**), suggesting greater photosynthetic capacity and enhanced response to distress signals and, ultimately, better metabolic flexibility to cope with their environmental conditions. Finally, we also measured the levels of anthocyanins in plants harvested in the three different sites (**Figure 1G**). As photoprotective agents, anthocyanins protect the photosynthetic machinery by absorbing excess UV and visible light, scavenging free radicals, reducing oxidative stress, and maintaining water homeostasis (Zhao et al., 2022). The levels of these protective pigments slightly increased from Site 1 to Site 3 plants, suggesting that the latter site is the most threatened by environmental stressors (Bertini et al., 2022).

### 3.2 Metatranscriptomic analysis

To delve deeper into the molecular mechanisms underlying the resistance and response of *C. quitensis* to different environmental factors, a comprehensive study was conducted using RNA-seq to highlight differentially expressed genes (DEGs) in *C. quitensis* plants collected from three different sampling sites. The total raw data were filtered, generating a mean of 80849415 high-quality reads, which represented 99% of total raw reads (**Supplementary Table 3**). The transcriptome sequencing was verified by quality control, as described in Section 2.6, and then sequencing data were aligned with the annotated *C. quitensis* transcriptome (Arthofer et al., 2015), making further analyses possible. In particular, the Principal Component Analysis (PCA) analysis cumulatively explained 53.8% of the total sample variance and also showed high distances between the three sites and a high reproducibility among the replicates (**Supplementary Figure 3**). By performing RNA-seq on *C. quitensis* leaves, we uncovered DEGs related to both the plant and its associated microbial community (**Supplementary Table 4**). The annotation process was performed using the NCBI genomic database, which enabled the identification of genes associated with Plants (94%), Bacteria (5.76%), Fungi (0.51%), other Eukaryotes (0.24%) and viruses (0.0032%) (**Supplementary Table 5**). For plant-related DEGs, we continued the analysis by performing a gene enrichment analysis, which allowed us to decipher the functional relevance of DEGs. For the plant-associated bacterial community, we carried out taxonomic analyses to highlight community structure and differential abundance analyses to highlight differentially abundant (DA) transcripts associated to specific bacterial strains across the three sites.

#### 3.2.1 Analysis of *C. quitensis* DEGs

**Figure 2:**
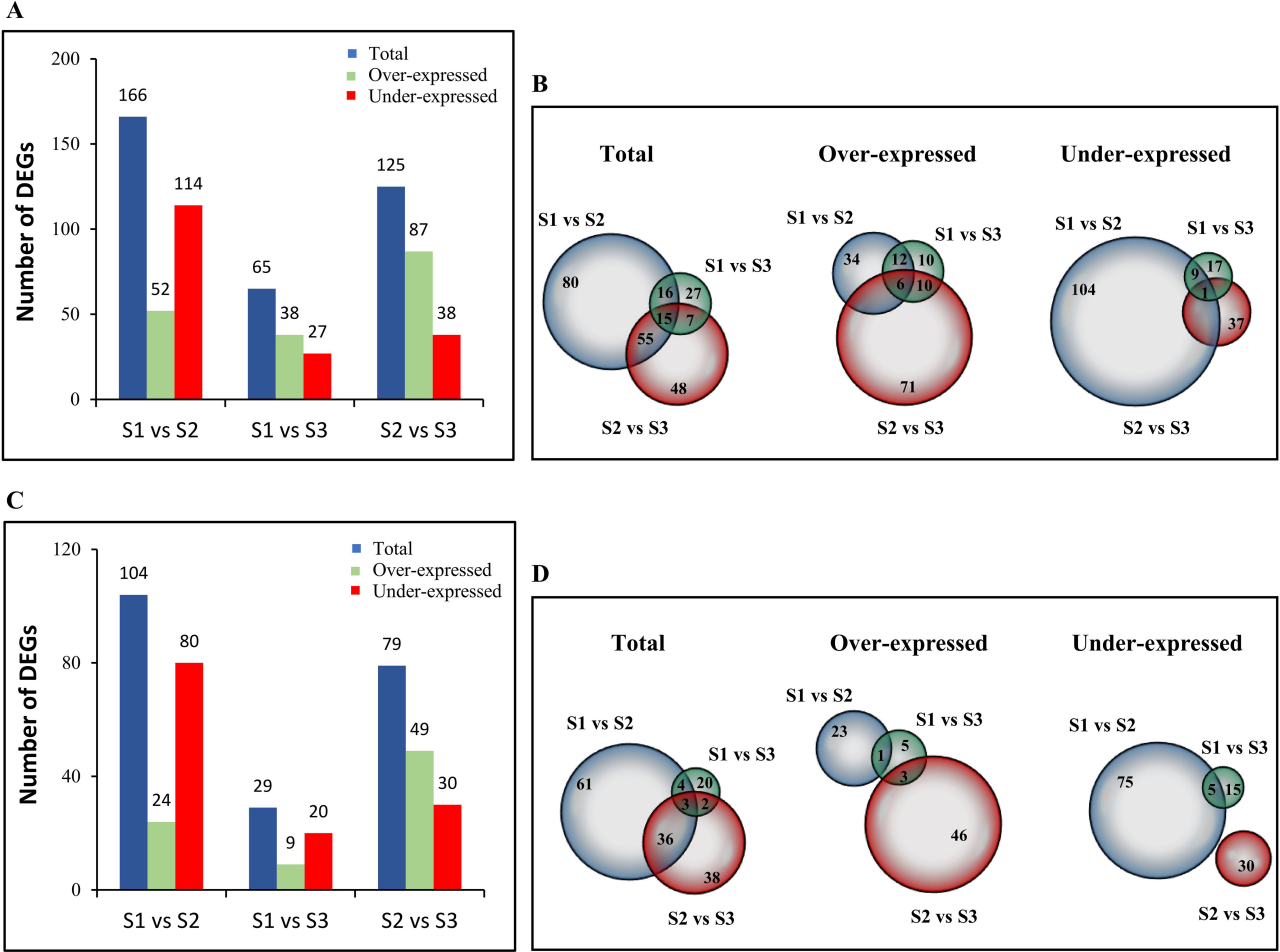
Numbers of *C. quitensis***(A)** and *Arabidopsis* putative TAIR orthologous **(C)** DEGs in different comparison groups. Venn diagrams showing the number of shared and unique *C. quitensis***(B)** and *Arabidopsis* putative TAIR orthologous **(D)** DEGs among comparisons. Numbers within each circle indicate the number of genes detected in that specific comparison. Overlapping regions represent genes shared between two or more comparisons, while non-overlapping regions indicate genes unique to a single comparison.

As for plant-annotated genes, 668 DEGs were disclosed comparing Site 1 vs Site 2, whereas 271 and 507 DEGs were highlighted comparing Site 1 vs Site 3 and Site 2 vs Site 3, respectively (**Supplementary Table 6**). To enhance the robustness of the functional analysis, an additional filtering step was conducted, selecting only DEGs with a sequence identity of 80% or higher to annotated plant sequences and removing all duplicates across all samples. Following this filtering, we identified 166 DEGs in the Site 1 vs Site 2 comparison, of which 52 DEGs were over’-expressed and 114 under-expressed (**Figure 2A**). Moving on the comparison between Site 1 vs Site 3, we identified 65 DEGs, among which 38 were found to be over-expressed and 27 under-expressed. Lastly, the Site 2 vs Site 3 comparison unveiled 125 total DEGs, being 87 over-expressed and 38 under-expressed. Overlap analysis using Venn diagrams highlighted unique and shared DEGs in each site comparison, underlining a core of shared genes that are likely to play a common role regardless of sampling sites (**Figure 2B**). These results suggest that gene expression can be programmed differently in response to varying environmental conditions at each site, which may enhance plants’ acclimation and effectiveness in dealing with local stressors.

To gain insight into the functional profile of the dataset, a gene enrichment analysis was performed. The limited availability of annotation data for non-model or less studied species represents a significant obstacle in pathway analysis and the selection of orthologous model species that could serve as a reliable proxy becomes useful (Pai et al., 2018). *Arabidopsis thaliana* has proved to be an ideal model organism, thanks to its comprehensive functional annotations and the availability of numerous bioinformatic tools that have allowed us to delve deep into gene functions, regulatory networks, and metabolic pathways (Bao et al., 2014). Despite the evolutionary gap between *Arabidopsis* and *C. quitensis*, the well-annotated *Arabidopsis* genome has provided us with a solid basis for identifying and comparing our DEGs. From our pool of 155 unique DEGs across all datasets, we identified a set of plant genes that exhibited the best alignment with *Arabidopsis* genes. This selection was based on stringent criteria, i.e., an e-value of ≤ 0.01 and a bit-score of ≥ 70, ensuring high confidence in the putative orthologous relationships. Through this accurate process, we successfully identified 119 unique genes that were deemed putative *Arabidopsis* orthologs of our DEGs. The distribution patterns of these genes showing differential abundance were analysed across the three distinct sampling sites (**Figure 2C**). In detail, we identified 104 DEGs (24 over-expressed and 80 under-expressed) in the Site 1 vs Site 2 comparison, 29 DEGs (9 over-expressed and 20 under-expressed) in the Site 1 vs Site 3 comparison, and 79 DEGs (49 over-expressed and 30 under-expressed) in the Site 2 vs Site 3 comparison. Furthermore, the unique and shared orthologous DEGs in the different site comparison are shown in the Venn diagrams shown in **Figure 2D**.

#### 3.2.2 Gene enrichment analysis on plant DEGs

**Figure 3:**
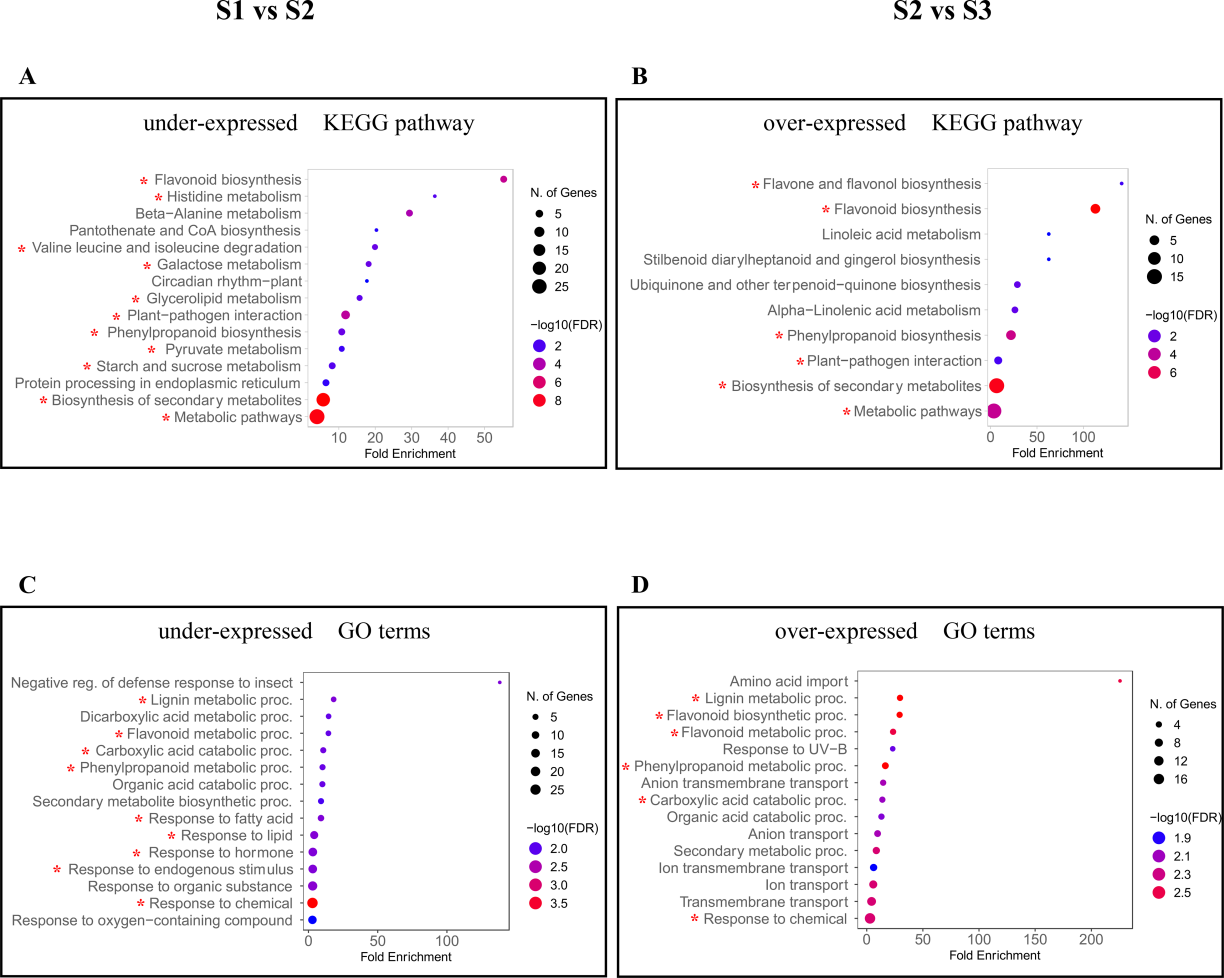
Enrichment analysis of orthologous DEGs in different comparison groups. KEGG pathways and GO terms under-expressed in the site 1 vs site 2 comparison **(A, C)** and over-expressed in the Site 2 vs Site 3 comparison **(B, D)** are represented. The size of the dots corresponds to the number of enriched genes, and dot colour reflects the –log10(FDR) value. The KEGG pathways and GO terms associated with the genes discussed in the text are marked with a red asterisk.

Previous proteomics study carried out on *C. quitensis* plants thriving in the same three sites revealed that the plant finely tunes its metabolic activity and stress response depending on the prevailing environmental conditions. Specifically, plants growing at Site 2 showed higher metabolic activity and resistance to abiotic and biotic stresses than those growing at other sites. Furthermore, they appeared to be particularly skilled in managing oxidative stress, also showing a more robust immune response against biotic stresses (Bertini et al., 2022). In the present study, we aimed to further investigate the effect of different environmental signals on the growth of *C. quitensis* to corroborate and possibly expand the previous analysis. Gene enrichment analysis is a powerful analytical approach for elucidating the functional relevance of DEGs and for uncovering the underlying biological processes they represent. The set of *Arabidopsis* orthologs served as an essential reference point for our analysis. We mainly focused on the over-expressed DEGs in Site 2 relative to the other sites, analysing the under-expressed DEGs in the comparison between the Site 1 vs Site 2 dataset (**Figures 3A, C**) and the over-expressed DEGs in the comparison between Site 2 vs Site 3 dataset (**Figures 3B, D**). In each pairwise comparison, gene enrichment analysis enabled the categorisation of under- and over-expressed DEGs into KEGG pathways and Gene Ontology (GO) terms (**Supplementary Table 7**).

Genes over-expressed at Site 2 were grouped into four major functional categories related to photosynthesis, catabolic processes in primary metabolism, phenylpropanoid secondary metabolism and response to stress.

#### 3.2.3 Photosynthesis-related genes

By analysing the over-expressed genes in the Site 2 vs Site 3 data set, we highlighted the *psaA* (ID: YP_009867265) and *psbA* (ID: YP_009867176) genes which are key components of the photosynthetic light reactions and are involved in the core structure and function of Photosystem I (PSI) and Photosystem II (PSII), respectively (Nelson and Yocum, 2006). The upregulation of these genes in Site 2 plants compared to the other sites reflects a site-specific transcriptomic signature associated with local microclimatic conditions, which support enhanced photosynthetic efficiency and electron transport stability. Moreover, among the over-expressed genes of the Site 2 vs Site 3 data set, we highlighted the *RBCL* (ID: YP_009867191) gene which encodes the catalytic subunit of RuBisCO (Ribulose-1,5-bisphosphate carboxylase/oxygenase), the key enzyme in the Calvin–Benson cycle that fixes atmospheric CO_2_ into organic carbon (Parry et al., 2013). An increase in *RBCL* transcript levels suggests a physiological adjustment toward maximising carbon assimilation capacity, possibly as a compensatory mechanism to support growth or metabolic activity under conditions where carbon acquisition might be limited. This is especially relevant in environments characterised by low temperatures, high irradiance, or short photoperiods, such as those inhabited by *C. quitensis*. In such settings, optimising the efficiency of RuBisCO becomes critical for sustaining biomass production and survival.

Overall, the concurrent overexpression of genes involved in the light reactions (e.g., *psaA* and *psbA*) and carbon fixation (e.g., *RBCL*) suggests coordinated transcriptional regulation across photosynthetic apparatus, likely reflecting a structural and functional adaptations that enhance light harvesting and carbon assimilation under harsh climatic conditions. This integrated response underscores the greater capacity of Site 2 plants, compared to those from the other sites, to fine-tune these pathways, thereby sustaining photosynthetic performance and metabolic homeostasis. Moreover, the higher Chl a/Car ratio observed in Site 2 plants further suggests functional adjustments that support metabolic balance and resilience under severe environmental conditions.

#### 3.2.4 Catabolic processes

##### 3.2.4.1 Carbohydrate metabolism

Comparative transcriptomic analysis between Site 1 and Site 2 revealed a widespread under-expression of genes related to carbohydrate and amino acid metabolism in Site 1, reinforcing the idea that plants at Site 2 exhibit a more active metabolic state (**Figures 3A, C**).

Within the Galactose metabolism pathway (**Figure 3A**), we detected the acid beta-fructofuranosidase 3 (*BFRUCT3*, ID: XP_010676174), also known as vacuolar invertase (VI1), an enzyme involved in the catalysis of the irreversible hydrolysis of sucrose into glucose and fructose and in the regulation of sucrose transport (Avigad, 1982). Its enhanced expression in Site 2 plants points to a potentially central role in supporting carbohydrate turnover under harsh environmental conditions.

In addition, *GOLS1* (ID: XP_021734496) and *AGAL3* (ID: XP_015575755), two key genes in raffinose family oligosaccharide (RFO) metabolism and known for their roles in sugar storage, phloem transport, and osmoprotection during stress (Sengupta et al., 2015; Sanyal et al., 2023), were identified as part of the transcriptomic signature of Site 2 plants. Notably, *GOLS1* was significantly over-expressed in Site 2 vs Site 3 within the GO category Response to chemical (**Figure 3D**), suggesting a stronger and more robust stress-related regulatory capacity in Site 2 plants. Its enrichment in Site 2 also compared to Site 1, further suggests a potential role for this gene in supporting a site-specific adaptive advantage. Similarly, *AGAL3*, which is involved in cell wall remodelling and hydrolysis of α-galactosyl residues, has been associated with developmental processes and stress sensitivity (Gu et al., 2018; Reyes-Pérez et al., 2019; Sun et al., 2023). Its upregulation in Site 2 dataset underlines the active modulation of structural and osmotic processes in Site 2 plants in response to their specific environmental context. Overall, this transcriptomic pattern reveals a site-specific transcriptional reprogramming likely sustaining the enhanced metabolic and stress-responsive traits observed in Site 2 plants and corroborates previous functional annotations by Bertini et al. (2022).Additionally, two genes involved in Starch and sucrose metabolism, i.e. β-glucosidase 40 (*BGLU40*, ID: XP_021718407) and β-amylase 3 (*BAM3*, ID: XP_021768081), were detected. Although these enzymes are classically associated with carbohydrate mobilisation, their functions extend beyond primary metabolism. Both have been implicated in stress adaptation and hormone signalling pathways (Monroe et al., 2014), suggesting a dual metabolic and regulatory role under environmental pressure. Notably, *BAM3* emerged as a particularly relevant gene in our analysis, as it was consistently over-expressed also in Site 2 vs Site 3 within the categories Biosynthesis of secondary metabolites and Metabolic pathways (**Figure 3B**). This site-specific upregulation positions *BAM3* as a candidate regulator of the enhanced stress-response capacity and metabolic flexibility observed in Site 2 plants. BAM3 is involved in starch degradation into maltose, a readily mobilizable energy source under stress conditions. In addition, starch-derived sugars act as osmoprotectants and signalling molecules that modulate stress-responsive gene expression. Therefore, the upregulation of *BAM3* in Site 2 plants likely reflects a strategic metabolic adjustment that enables efficient remobilisation of energy reserves and activation of defence responses. Together, this finding provides transcriptomic evidence for a more dynamic and adaptive carbohydrate metabolism in Site 2 plants, potentially underpinning their greater resilience compared to plants from Site 1 and Site 3.

##### 3.2.4.2 Redox homeostasis

Among the under-expressed genes in the Site 1 vs Site 2 dataset, we observed a significant enrichment of genes involved in Glycerolipid metabolism and amino acid metabolism (specifically, Histidine metabolism and Valine, Leucine, and Isoleucine degradation pathways) (**Figure 3A**), such as two aldehyde dehydrogenase (ALDH) genes, *ALDH2B7* (ID: XP_010682575) and *ALDH3I1* (ID: XP_021863785). These genes belong to a conserved family of NAD(P)+-dependent enzymes that detoxify reactive aldehydes by converting them into non-toxic carboxylic acids (Singh et al., 2013) and play a key role in mitigating oxidative stress (Zhao et al., 2017). Their over-expression in Site 2 plants underlines a molecular distinction between Site 1 and Site 2 plants in managing redox homeostasis, indicating a reduced capacity for aldehyde detoxification in Site 1 plants, which may translate to lower resilience to oxidative stress. In the same dataset, we also identified the NADP-dependent malic enzyme (*NADP-ME4*, ID: XP_021729206), within the Pyruvate metabolism pathway (**Figure 3A**) and the Carboxylic acid metabolic process (**Figure 3C**). NADP-ME4 catalyses the decarboxylation of malate to pyruvate and NAD(P)H, contributing not only to central carbon metabolism but also to the production of reducing power essential for the biosynthesis of secondary metabolites, such as flavonoids and lignin, which are known to play protective roles in stress response (Sun et al., 2019). This pattern is consistent with the broader trend of reduced expression of metabolic and stress-responsive genes in Site 1, suggesting that Site 2 plants activate more robust protective mechanisms that may contribute to their superior performance under extreme Antarctic conditions.

##### 3.2.4.3 Carbon–nitrogen metabolism and GABA signalling

Additionally, among the under-expressed genes in the Site 1 vs Site 2 comparison, we identified *FDH1* (ID: XP_023926463), encoding formate dehydrogenase 1, an NAD^+^-dependent enzyme that catalyses the oxidation of formate to carbon dioxide, within the Metabolic pathways category (**Figure 3A**). Given the established role of FDH1 in one-carbon metabolism and plant stress responses (Alekseeva et al., 2011; Marzorati et al., 2021), its reduced expression in Site 1 plants suggests a diminished engagement of these processes, further supporting the idea that plants at Site 2 maintain a more metabolically active and stress-responsive profile. Within the same KEGG pathway, we also uncovered *GAD1* (ID: XP_021753174), encoding glutamate decarboxylase 1, which converts intracellular glutamate into γ-aminobutyric acid (GABA), a multifunctional metabolite involved in stress signalling and carbon-nitrogen balance (Tzin and Galili, 2010). Notably, *GAD1* was also found over-expressed in the Site 2 vs Site 3 dataset (**Figure 3B**), further supporting the view that GABA biosynthesis is particularly enhanced in Site 2 plants. This consistent pattern across comparisons points to a site-specific upregulation of *GAD1* and highlights its likely involvement in a broader adaptive mechanism that supports plant resilience under extreme environmental conditions.

Collectively, these transcriptional signatures point to a coordinated metabolic reprogramming in Site 2 plants, in which carbohydrate turnover, redox buffering, and carbon–nitrogen balance converge to support enhanced acclimation capacity. This aligns with previous proteomic data by Bertini et al. (2022) showing enhanced metabolic activity at this site and underscores the role of transcriptional regulation in shaping the acclimation strategies of *C. quitensis* in the Antarctic environment.

#### 3.2.5 Phenylpropanoid pathway

##### 3.2.5.1 Shikimate pathway and aromatic amino acid biosynthesis

**Figure 4:**
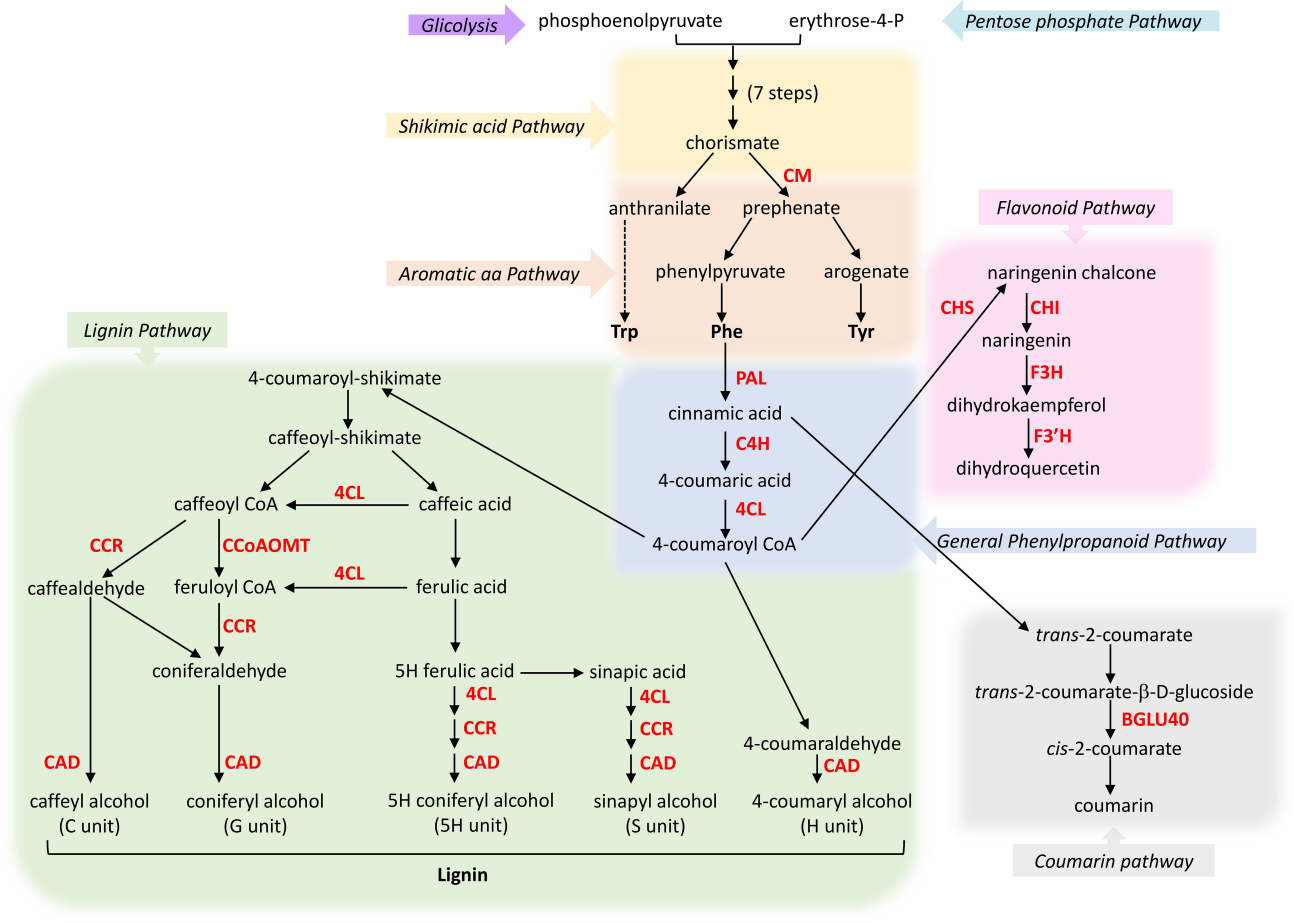
Diagram of the phenylpropanoid pathway leading to lignin, flavonoid and coumarin biosynthesis. Enzymes indicated in red are encoded by genes identified during the metatranscriptomic analysis and discussed in the text. CYP75B1 is required for full F3’H activity. Enzymes involved in the Aromatic aa and General Phenylpropanoid pathways include: chorismate mutase (CM), phenylalanine ammonia-lyase (PAL), cinnamic acid 4-hydroxylase (C4H), and 4-coumaroyl-CoA ligase (4CL). Flavonoid pathway: chalcone synthase (CHS), chalcone isomerase (CHI), flavanone 3-hydroxylase (F3H) and flavonoid 3’ hydroxylase (F3’H). Coumarin pathway: β-glucosidase 40 (BGLU40). Lignin pathway: cinnamoyl-CoA reductase (CCR), cinnamyl alcohol dehydrogenase 4 (CAD4), and caffeoyl CoA O-methyltransferase (CCoAOMT).

The shikimate pathway is a central metabolic route in plants that drives the biosynthesis of the aromatic amino acids - phenylalanine (Phe), tyrosine (Tyr), and tryptophan (Trp) - which serve as precursors for a broad array of secondary metabolites, including flavonoids, lignins, and other phenolic compounds (**Figure 4**) (Tzin and Galili, 2010). Such metabolites play essential roles in plant development, adaptation, and defence, particularly in response to abiotic stresses such as drought, salinity, and UV radiation (Dong and Lin, 2021). Notably, recent studies have shown that the accumulation of flavonoids like kaempferol and quercetin contributes to ROS scavenging and stress mitigation (Jan et al., 2022; Iqbal et al., 2023).

Within this framework, our transcriptomic analysis provides novel insights into the transcriptional regulation of secondary metabolite biosynthetic pathways in *C. quitensis* across different environmental conditions. By comparing under-expressed genes in the Site 1 vs Site 2 dataset (**Figures 3A, C**) and the over-expressed genes in the Site 2 vs Site 3 dataset (**Figures 3B, D**), we highlighted several genes categorised in the KEGG pathway Biosynthesis of secondary metabolites (**Figure 3A**) and in the GO term Response to chemical (**Figure 3C**), many of them involved in the synthesis of phenolic compounds. Among these is *CM1* (ID: XP_038905076), encoding chorismate mutase 1, which catalyses the conversion of chorismate to prephenate, a key step directing carbon flux toward phenylalanine biosynthesis (**Figure 4**).

##### 3.2.5.2 General phenylpropanoid pathway

In the same datasets, we identified several genes of the general phenylpropanoid pathway (Phenylpropanoid biosynthesis, **Figure 3B**), as phenylalanine ammonia-lyase (*PAL1*, ID: XP_021730766), which represents the first enzyme of the route and is involved in the conversion of Phe to cinnamic acid. Subsequently, we disclosed cinnamic acid 4-hydroxylase (C4H), which catalyses the transformation of cinnamic acid in 4-coumaric acid, which, in turn, is converted to 4-coumaroyl CoA by 4-coumaroyl-CoA ligases 3 (4CL3). The initial three steps of the general phenylpropanoid pathway, catalysed by PAL1, C4H, and 4CL, are essential for the formation of phenylpropanoid monomers, which serve as the building blocks for all other phenolic compounds.

##### 3.2.5.3 Coumarin biosynthesis

As a side branch of the general phenylpropanoid pathway, the coumarin route produces coumarins starting from cinnamic acid (**Figure 4**). Interestingly, we found the gene β-glucosidase 40 (*BGLU40*, ID: XP_021718407) involved in the biosynthesis of coumarins, which are hydroxycinnamic phenolic acids that mediate physiological processes in plants at different growth and developmental stages. Besides their role in increasing plant tolerance to salinity, coumarins are also involved in strengthening antioxidant defence having also direct antifungal and antibacterial properties that play significant roles in plant disease resistance (Stringlis et al., 2018).

##### 3.2.5.4 Structural and antioxidant specialisation: flavonoid and lignin branches

In addition to the coumarin route, the general phenylpropanoid pathway also branches into flavonoid pathway and lignin pathway, leading to the formation of either flavonoids or lignin, respectively (**Figure 4**). In this study, among the under-expressed genes in the Site 1 vs Site 2 comparison, we identified four genes involved in the flavonoid pathway (*CHS*, *CHI*, *F3H* and *F3’H*), categorised in the Flavonoid biosynthesis KEGG pathway (**Figure 3A**) and in the Flavonoid metabolic processes and Phenylpropanoid metabolic processes GO terms (**Figure 3C**), as well as three genes involved in the lignin branch (*CCoAOMT1*, *CCR1* and *CAD4*), categorised within the GO term Lignin metabolic process (**Figure 3C**). Following the flavonoid branch, 4-coumaroyl CoA is transformed into naringenin chalcone by chalcone synthase (CHS or TT4, ID: XP_021837196), which seems to be the key enzyme for flavonoid production, catalysing the first committed step in the flavonoid branch. Naringenin chalcone is isomerised by chalcone isomerase (CHI, ID: XP_021758232) to produce the flavanone naringenin, which is further transformed into dihydrokaempferol by flavanone 3-hydroxylase (F3H, ID: XP_006403939). Subsequently, dihydrokaempferol is converted into dihydroquercetin by flavonoid 3’ hydroxylase (F3’H, ID: XP_010685305). On the other hand, following the lignin branch, 4-coumaroyl-CoA initiates the lignin pathway leading to the formation of the basic units of natural lignin polymers consisting of C, G, 5H, S and H units (**Figure 4**) (Tzin and Galili, 2010). Among the Site 1 vs Site 2 under-expressed genes, we found genes coding caffeoyl CoA O-methyltransferase 1 (*CCoAOMT1*, ID: XP_010690616), cinnamoyl-CoA reductase 1 (*CCR1*, ID: XP_022714742) and cinnamyl alcohol dehydrogenase 4 (*CAD4*, ID: XP_010691671), which produce the monolignols constituting the building blocks of lignin, acting sequentially from caffeoyl CoA. Notably, some of these genes were also over-expressed in the Site 2 vs Site 3 comparison, both in KEGG pathways and GO terms.

##### 3.2.5.5 Monolignol transport and lignin polymerisation

Although the mechanism underlying monolignol transport from the cytosol to the cell wall remains not completely understood, ABC transporters have been proposed as potential contributors to this process (Perkins et al., 2019). In this context, two genes, *ABCB11* (ID: XP_010688391) and *ABCG6* (ID: XP_021850035), both encoding ABC transporters, were identified among the under-expressed genes in the Site 1 vs Site 2 dataset. Although their direct involvement in monolignol transport has yet to be demonstrated, their expression patterns suggest a possible role in the regulation, trafficking or cellular localisation of phenylpropanoid-derived monolignols. Following transport to the cell wall, monolignols must undergo oxidative polymerisation to form lignin, a process primarily mediated by peroxidases and/or laccases (LACs) (Zhao et al., 2013). Among these enzymes, *LAC5* (ID: XP_021732559) has been specifically implicated in the biosynthesis of G lignin and neolignans in *Arabidopsis* (Yonekura-Sakakibara et al., 2021). Notably, *LAC5* was found to be under-expressed in the Site 1 vs Site 2 dataset and over-expressed in the Site 2 vs Site 3 dataset, suggesting that this laccase may contribute to lignin polymerisation dynamics even in *C. quitensis*.

##### 3.2.5.6 Transcriptional regulation of the phenylpropanoid pathway

Phenylpropanoid metabolism is regulated by a complex network of signalling pathways and regulatory mechanisms, including transcriptional, epigenetic and post-translational controls, as well as phytohormone signalling. Among these layers of regulation, transcriptional control accounts for the majority of regulatory effects and is crucial in controlling the production of phenylpropanoid-derived metabolites (Yuan and Grotewold, 2020). At the transcriptional level, the phenylpropanoid pathway is primarily controlled by MYB-type transcription factors (TFs), ternary complexes (MBW) formed by MYB, basic helix-loop-helix (bHLH), and WD40 proteins, as well as other TFs (Liu et al., 2015). In our study, we identified two genes encoding TFs belonging to the MYB family, specifically *MYB15* (ID: XP_010533288) and *MYB24* (ID: XP_027073189). *MYB15* was detected among the over-expressed DEGs in the Site 2 vs Site 3 comparison, whereas *MYB24* was under-expressed in Site 1 vs Site 2 dataset and the over-expressed in the Site 2 vs Site 3 dataset. It has been reported that MYB15 controls lignin biosynthesis in *C. morifolium* and *A. thaliana* (Chezem et al., 2017; An et al., 2019) and stimulates the production of G lignin by directly binding to the AC-rich elements in the promoter region of phenylpropanoid pathway genes, such as *PAL1*, *C4H*, and *COMT* (Tzin and Galili, 2010). Furthermore, MYB15 has been also reported to regulate the expression of the stilbene synthase (*STS*) gene (Höll et al., 2013), which catalyse the formation of the stilbene skeleton starting from cinnamoyl CoA or 4-coumaroyl CoA. Notably, 4-coumaroyl CoA represents a central metabolic intermediate that links multiple branches of the phenylpropanoid pathway, including those leading to the biosynthesis of flavonoids, lignin, and stilbenes, small non-flavonoid polyphenols endowed with several helpful roles in plant defence against both biotic and abiotic stress (Gao et al., 2024). Similarly, MYB24 has been reported to control lignin biosynthesis by activating the expression of *4CL1* and *CCoAMT1* in pear fruit stone cells (Xue et al., 2023). Beyond its role in phenylpropanoid metabolism, MYB24 is also involved in the regulation of very-long chain fatty acid biosynthesis during hypersensitive response in *Arabidopsis* (Raffaele et al., 2008).

In summary, the phenylpropanoid pathway is essential for plant survival, contributing not only to their growth and development but also to defence against environmental stresses and pathogen attacks (Dong and Lin, 2021). Overall, the coordinated over-expression of key genes across the shikimate, general phenylpropanoid, flavonoid, and lignin branches indicate enhanced metabolic flux toward phenolic compound biosynthesis in Site 2 plants. This transcriptional reprogramming likely supports structural reinforcement, antioxidant capacity, and defence readiness, collectively contributing to improved acclimation to the environmental conditions characterising this site.

#### 3.2.6 Stress response

##### 3.2.6.1 Stress signal transduction

Data mining of our dataset revealed several DEGs associated with plant defence responses to both biotic and abiotic stimuli. A central and widely conserved component of these responses is the regulation of reactive oxygen species (ROS). Although excessive ROS accumulation disrupts cellular redox homeostasis and causes oxidative damage, low and tightly controlled ROS levels act as essential signalling molecules (Mittler et al., 2011; Mittler, 2017). In plants, ROS function as cellular second messengers that coordinate both growth and development, as well as a rapid response to environmental clues (Foyer, 2021). This signalling is largely mediated by plant-specific NADPH oxidases, known as respiratory burst oxidase homologs (RBOHs), which regulate the trade-off between defence and growth (Marino et al., 2012; Kaya et al., 2019). In *Arabidopsis*, RBOHs contribute to ROS generation during pathogen-associated molecular pattern-triggered immunity (PTI) and effector-triggered immunity (ETI) responses (Li et al., 2014), and they also play crucial roles in the response of plants to abiotic stresses such as drought, salt and extreme temperatures (Liu et al., 2020, Liu et al., 2022; Zhang et al., 2022).

Consistently, within the Plant-pathogen interaction pathway identified in both the Site 1 vs Site 2 (**Figure 3A**) and Site 2 vs Site 3 (**Figure 3B**) data sets, we found *RBOHE* (ID: XP_021756720), which has been reported to be involved in plant growth and root architectural changes during the *Arabidopsis*-*Trichoderma* interaction (Esparza-Reynoso et al., 2023). Within the same KEGG pathway (**Figure 3A**), we also disclosed three genes encoding for Calcium-dependent protein kinases (CPKs) which are essential regulators of plant responses to osmotic stress (*CPK4*, ID: XP_021767245) (Fan et al., 2023), ABA-mediated stomatal signalling (*CPK9*, ID: XP_010675716) (Chen et al., 2019) and salt-responsive root growth in *Arabidopsis* (*CPK16*, ID: XP_009590568) (Silamparasan et al., 2023).

##### 3.2.6.2 Heat shock proteins and pathogen-responsive genes

The overall picture of enhanced stress protection exhibited by plants at Site 2 is further supported by the overexpression of HSP90-3 (ID: XP_023878992), a cytosolic heat shock protein involved in the activation of defence responses and cell death under chilling stress (Bao et al., 2014, Lu et al., 2022). In addition, we disclosed two defence-related genes associated with hormone-mediated immunity, namely *CAP* (Cys-rich secretory proteins, Antigen5, Pathogenesis-Related 1 super family, ID: XP_021776323), categorised within the Plant-pathogen interaction pathway (**Figures 3A, B**) and *HEL* (PR4, ID: XP_0217422609, associated to the Response to chemical GO term (**Figure 3D**). *CAP* is mainly induced by biotrophic pathogens, which causes the increase of salicylic acid (SA) leading to the onset of systemic acquired resistance (SAR), while *HEL* is predominantly over-expressed upon necrotrophic pathogens attack that use jasmonic acid (JA) as a signal molecule contributing to local acquired resistance (LAR) (Ali et al., 2018).

##### 3.2.6.3 Hormone and chemical response genes

In agreement with the above, several genes categorised in the Response to hormone/endogenous stimulus or more generally in the Response to chemicals GO terms were found under-expressed in the Site 1 vs Site 2 comparison (**Figure 3C**). These include genes encoding for: (i) Acyl activating enzyme3 (*AAE3*, ID: XP_034711383), in which contribute to defence against fungal pathogens due to its role in the catabolism of oxalate, produced as a phytotoxin to aid host infection (Foster et al., 2016); (ii) 1-aminocyclopropane-1-carboxylate oxidase 4 (*ACO4 or EFE*, ID: XP_021766818), which acts downstream of 1-aminocyclopropane-1-carboxylate synthase (*ACS*), to catalyse ethylene biosynthesis, essential for numerous physiological processes related to growth and development, but also to tolerance and survival under multiple stress conditions (Khan et al., 2024); (iii) acid beta-fructofuranosidase 3 (*BFRUCT3*), a key enzyme in carbohydrate metabolism (as discussed, in the Catabolic processes paragraph) that also regulates cellular osmotic balance and, more generally, contributes to plant responses to environmental stress (Ruan et al., 2010); (iv) calreticulin 3 (*CRT3*, ID: XP_031402499), which is an endoplasmic reticulum (ER) calcium binding protein involved in a multitude of different tasks in addition to its activity as a molecular chaperone. CRT3-mediated signalling is closely connected with phytohormones-dependent pathways that are crucial for preserving innate immunity and protecting plants from bacterial and fungal diseases (Joshi et al., 2019); (v) lipoxygenases (*LOXs*, ID: XP_010673986), encoding enzymes that catalyse the conversion of polyunsaturated fatty acids into fatty acid hydroperoxides, which are key intermediates in the octadecanoid signalling pathway. They play a pivotal role in various biological processes, including the production of JA, and response to biotic (insect invasions, pest infestations, and pathogen attacks) as well as abiotic stresses (wounding, exposure to UV radiation, extreme temperatures, oxidative damage, and drought) (Singh et al., 2022).

Collectively, plants at Site 2 exhibit a coordinated stress-responsive profile, which likely enhance their resilience to the various stresses they encounter. This transcriptional pattern is consistent with previously reported proteomic evidence (Bertini et al., 2022) and supports the notion that plants at Site 2 are molecularly equipped for enhanced resilience.

#### 3.3 Analysis of bacterial community associated to *C. quitensis*

**Figure 5:**
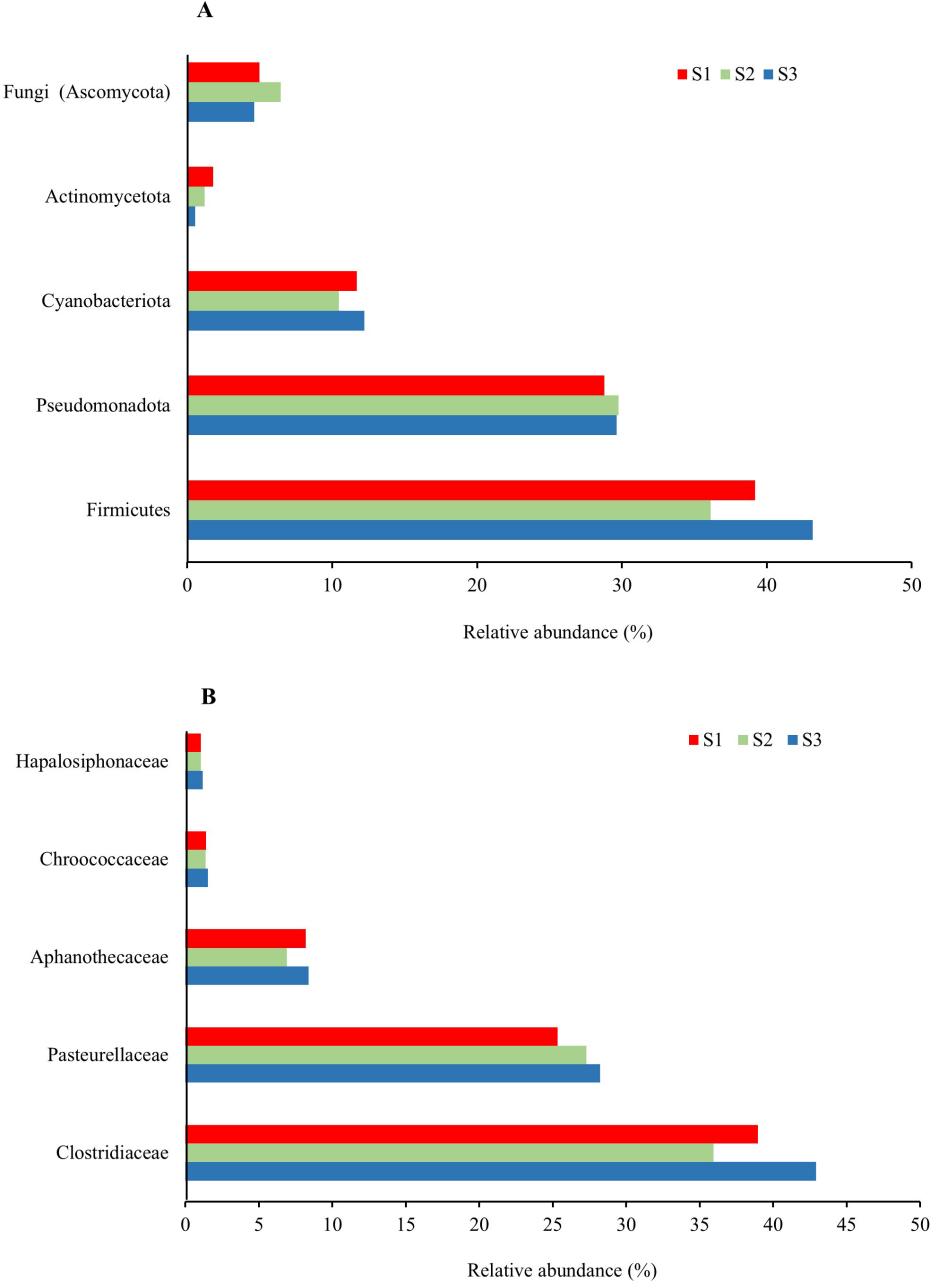
Relative abundance (%) of the most represented fungal and bacterial phyla **(A)** and bacterial families **(B)** across the three sampling sites, inferred from sequence similarity of the most abundant transcript groups. For each site the number of replicates is n=3.

To exploit metatranscriptomic data for putative taxonomic assignments, sequencing reads were filtered and processed as described in Section 2.7. Reads classified as “passed” in the Genome database, ranging from 71476348 to 87303437 depending on the sampling site, were used for taxonomic analysis (**Supplementary Figure 4**, **Supplementary Table 8**). Nevertheless, we acknowledge that taxonomic inferences derived from metatranscriptomic data should be interpreted with caution. Indeed, unlike DNA-based approaches, metatranscriptomics captures only the actively expressed fraction of the community, which can vary across tissues, developmental stages, environmental conditions, and sequencing depth. For these reasons, the taxa discussed below should be regarded as putative indicators of metabolically active community members inferred from sequence similarity, rather than as confirmed organismal abundance or presence. To partially mitigate annotation-related bias, we focused our taxonomic analysis on bacterial communities, as bacterial transcripts are generally more abundant and better represented in reference databases than fungal or archaeal ones, allowing for more reliable taxonomic assignments. A total of 283 putative species-level assignments were inferred, displaying different relative transcript abundances across all samples (**Supplementary Table 9**). The top 40 taxa associated with the highest transcript abundance are listed in **Supplementary Table 10**, together with their classification into Phylum and Family. Between them, the most abundant bacterial phyla were Firmicutes, Pseudomonadota, Cyanobacteriota and Actinomycetota, while the Fungi Kingdom was predominantly represented by Ascomycota (**Figure 5A**). In particular, Firmicutes were more abundant at Site 3 (43,17%) and Site 1 (39,17%) than at Site 2 (36,12%) whereas Pseudomonadota were similarly represented at Site 2 and Site 3 (29,65% and 29,77%, respectively) and only slightly less abundant at Site 1 (28,80%). Recently, a taxonomic and functional survey of plant-associated microbial community in cold environments (alpine, Arctic and Antarctica) was conducted. Several studies have reported that Bacteroidota, Pseudomonadota, Firmicutes and Actinomycetota as the dominant bacterial phyla in some Arctic plants regardless of plant organs (Nissinen et al., 2012; Kumar et al., 2017; Given et al., 2020), although Pseudomonadota and Bacteroidota were reported to be predominant in the roots and Firmicutes in the leaves of *Oxyria digyna* (Given et al., 2020). These findings support the concept of plant tissue-specific bacterial communities in Arctic environment, as widely observed in temperate ecosystems (Li et al., 2022). Moreover, the same phyla were reported to be predominantly associated with rhizosphere, roots and leaves of the two endemic Antarctic plants *C. quitensis* and *D. antarctica* (Molina-Montenegro et al., 2019; Silva et al., 2020; Zhang et al., 2020). Our results (**Figure 5A**) are consistent with the existing literature and provide new insights into the microbial community associated with *C. quitensis*. At the family level (**Figure 5B**), Clostridiaceae and Pasteurellaceae, belonging to the Firmicutes and Pseudomonadota phyla, respectively, were the most predominant, whereas Aphanothecaceae, Chroococcaceae and Hapalosiphonaceae, all belonging to the Cyanobacteriota phylum, were less abundant. In agreement with the lower abundance of Firmicutes and Cyanobacteriota in Site 2 compared to the other sites (**Figure 5A**), the Clostridiaceae and Aphanothecaceae families were also less represented in Site 2 than in the other sites.

**Figure 6:**
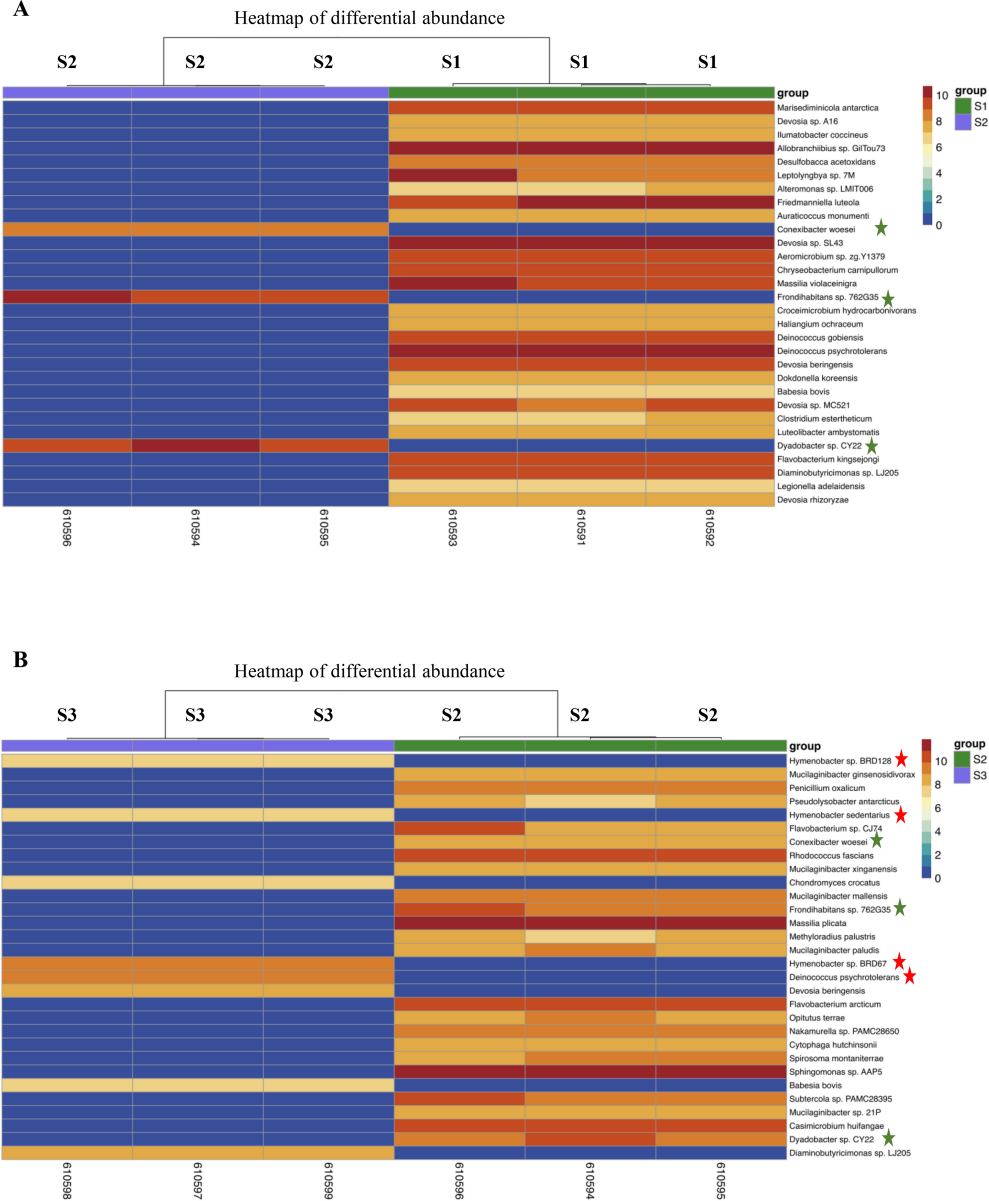
Heatmaps of the top 30 putative differentially abundant taxa in the pairwise comparison between site 1 vs site 2 **(A)** and site 2 vs site 3 **(B)**. Green stars indicate species more abundant in Site 2 with respect to both Site 1 and Site 3. Red stars indicate the taxa already described in Antarctica found as less abundant Site 2 vs Site 3 comparison.

To further characterise the *C. quitensis*-associated microbiota, a PCA was carried out to compare samples and assess intra- and inter-group clustering. The first two dimensions of the PCA analysis explained 70% of the total variance in the samples and highlighted differences between the three sites, also demonstrating high reproducibility among biological replicates within each site (**Supplementary Figure 5A**). Alpha diversity analysis indicated that the local environmental conditions at the three study sites may influence within-sample microbial diversity, as estimated using Faith’s phylogenetic diversity (PD) metric (**Supplementary Figure 5B**) (Faith, 1992). The higher phylogenetic diversity observed at Site 1 may be associated with the observed greater vegetation cover, which can promote niche diversification and microbial coexistence. Conversely, the sparse vegetation and potentially more extreme abiotic conditions at Site 3 may impose stronger selective pressures, resulting in reduced phylogenetic diversity. The intermediate diversity observed at Site 2 is consistent with transitional environmental characteristics between the two extremes. Beta diversity analysis based on four different metrics (Jaccard, Bray-Curtis, Weighted-UniFrac and Unweighted-UniFrac), revealed no significant separation among sites, suggesting broadly similar overall community composition across the sampling locations (**Supplementary Figure 6**). Despite the lack of global compositional differences among sites, we investigated whether specific taxa exhibited differential abundance patterns. The top 30 DA taxa identified in the pairwise comparison between Site 1 vs Site 2 and Site 2 vs Site 3 are shown in **Figure 6**. Given the limited functional characterisation of many Antarctic plant-associated microorganisms, interpreting the ecological relevance of all identified DA taxa remains challenging. The scarcity of genomic resources and experimental validation for many cold-adapted taxa restricts our ability to infer their specific roles in plant fitness, stress tolerance, or nutrient cycling. Therefore, rather than exhaustively reporting all possible comparisons, we focused our analysis on taxa that were significantly enriched in Site 2 relative to both Site 1 and Site 3, as these may represent distinctive microbial signatures associated with Site 2 plants. In addition, we prioritised taxa that have been previously described in Antarctic ecosystems, as these may hold ecological relevance supported by prior evidence. Among the DA taxa over-represented in Site 2 compared to both Site 1 and Site 3, *Conexibacter woesei, Frondihabitans* sp. 762G35 and *Dyadobacter* sp. CY22 were detected (**Figure 6A**). Members of the genus *Conexibacter* are strictly aerobic bacteria belonging to the Actinomycetota phylum and have been identified in soils and a wide range of environments, including Arctic ecosystems, through culture-dependent and metagenomic approaches (Monciardini et al., 2003; Pukall et al., 2010; Krivushin et al., 2015). Noteworthy, *C. woesei* has been described as a nitrate-reducing bacterium, suggesting a potential involvement in nitrogen cycling within plant-microbe association. The genus *Frondihabitans* has been described in plant environments, including the phyllosphere, and has been reported as a potential indicator genus of leaf-associated microbial communities. In particular, *Frondihabitans* has been identified on the leaves of both apple plants growing in the orchard environment (Longa et al., 2022) and *Arabidopsis* (Reisberg et al., 2013). Its recurrent association with plant leaves suggests potential ecological roles, including possible involvement in nutrient cycling, interplay with plant metabolism, and broader plant-microbe interactions (Zhang and Xu, 2007). The presence of *Dyadobacter* sp.CY22, belonging to the Bacteroidota phylum, has been highlighted in a range of temperate and cold environments, underlining the psychrotolerant nature of these microorganisms (Ma et al., 2023; Chaudhary et al., 2020). Among the DA taxa previously reported in Antarctica and known for their psychrotolerant characteristics, members of the genus *Hymenobacter* (Hirsch et al., 1998) and *Deinococcus psycrotolerans* (Tian et al., 2019), were identified as less abundant in the Site 2 vs Site 3 comparison (**Figure 6B**). However, current literature provides limited insight into their association with plants. Therefore, further functional characterisation is necessary to elucidate their potential role in association with *C. quitensis*.

## 4 Conclusions

In this study, we showed that *C. quitensis* is highly sensitive to even minor environmental changes and can reprogram its metabolic activity accordingly. Although the extreme logistical and environmental constraints inherent to Antarctic fieldwork limited climatic data collection at Site 3, the consistency of plant responses across independent physiological and structural traits supports the reliability of the site-specific comparisons. Along the coastal-to-inland gradient, Site 2 emerged as the most favourable microenvironment for *C. quitensis* growth and defence, integrating limited sea-spray exposure, balanced soil water availability, and reduced influence from the nearby glacier. Indeed, plants thriving at Site 2 exhibited structural and physiological adaptations that enhance light harvesting capacity, as indicated by a lower Chl a/b ratio and the overexpression of several photosynthesis-related genes. In addition, the upregulation of genes involved in active metabolism suggests a strong capacity to sustain growth-promoting pathways, thereby enhancing resilience under extreme environmental conditions (**Figure 3**). However, plant survival relies on a fine balance between growth and defence. In Site 2 plants, the phenylpropanoid pathway was particularly active, leading to the accumulation of defence-related secondary metabolites, including lignins, flavonoids, and quercetins (**Figure 4**), which play key role in mitigating oxidative stress. Additionally, several defence-related genes were overexpressed, including NADPH oxidases, which are involved in responses to drought, salinity, and extreme temperature, as well as PR1 and PR4, markers of salicylic acid- and jasmonic acid-mediated defence pathways. We also explored the *C. quitensis* leaf-associated microbial community, providing an initial view of the transcriptionally active microbial signatures associated with this Antarctic vascular plant. While these data do not allow definitive taxonomic or functional assignments, they highlight a set of putative, metabolically active microorganisms whose gene expression patterns are associated with *C. quitensis* leaves under natural conditions. Although the specific functional roles of these microorganisms remain to be elucidated, their inferred association with *C. quitensis* suggests a potential contribution to plant resilience and responsiveness to environmental cues. Future efforts aimed at isolating and culturing these microorganisms under controlled conditions will be essential to validate these associations and to uncover their physiological and metabolic capabilities, with possible applications in agriculture. In conclusion, this work demonstrates that even subtle environmental differences can significantly influence plant molecular responses, modulating key mechanisms involved in both growth and defence.

## Data Availability

The datasets presented in this study can be found in online repositories. The names of the repository/repositories and accession number(s) can be found in the article/**Supplementary Material**.
