# Supplementary material for: Exploring the molecular landscape of environmental responses in the Antarctic plant Colobanthus quitensis: insights from metatranscriptomic analysis
Source: Front Plant Sci. 2026 Mar 16;17:1774223. doi: 10.3389/fpls.2026.1774223 (PMC13034057; doi:10.3389/fpls.2026.1774223)
Supplement: Supplementary file 1 [file Image1.pdf]

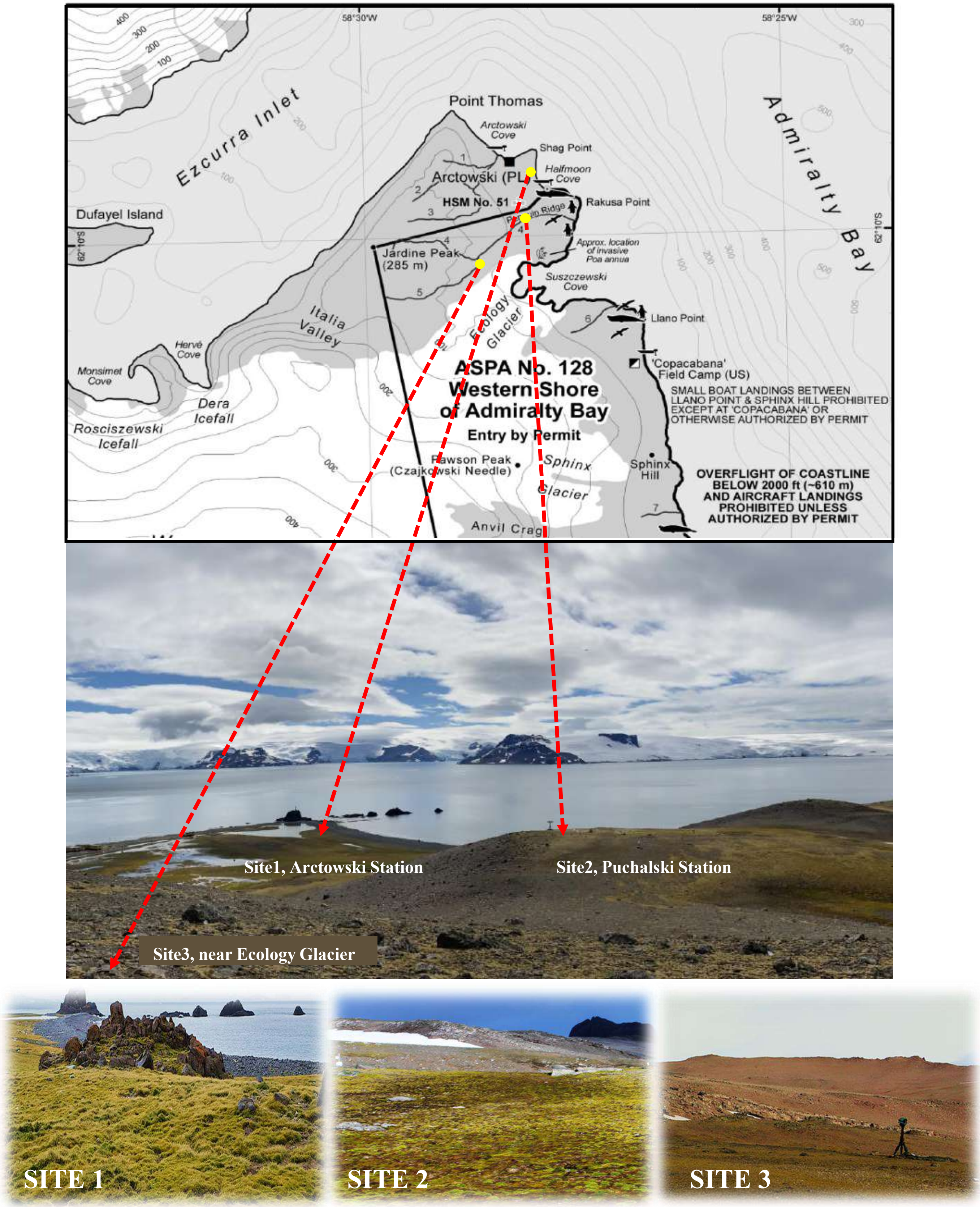

Figure S1. The sampling sites are located near the Polish Antarctic base H. Arctowski overlooking the Admiralty Bay on King George Island (Maritime Antarctica). Site 1 (S1, Arctowski Station) is on the seashore, Site 2 (S2, Puchalski Station) is located on a hill (50 m), and Site3 (S3) at an even higher altitude (60 m), near to the Ecology Glacier. Photo on the bottom by the author C. Caruso, on behalf of the National Program for Antarctic Research (PNRA).
