# Supplementary material for: Exploring the molecular landscape of environmental responses in the Antarctic plant Colobanthus quitensis: insights from metatranscriptomic analysis
Source: Front Plant Sci. 2026 Mar 16;17:1774223. doi: 10.3389/fpls.2026.1774223 (PMC13034057; doi:10.3389/fpls.2026.1774223)
Supplement: Supplementary file 2 [file Image2.pdf]

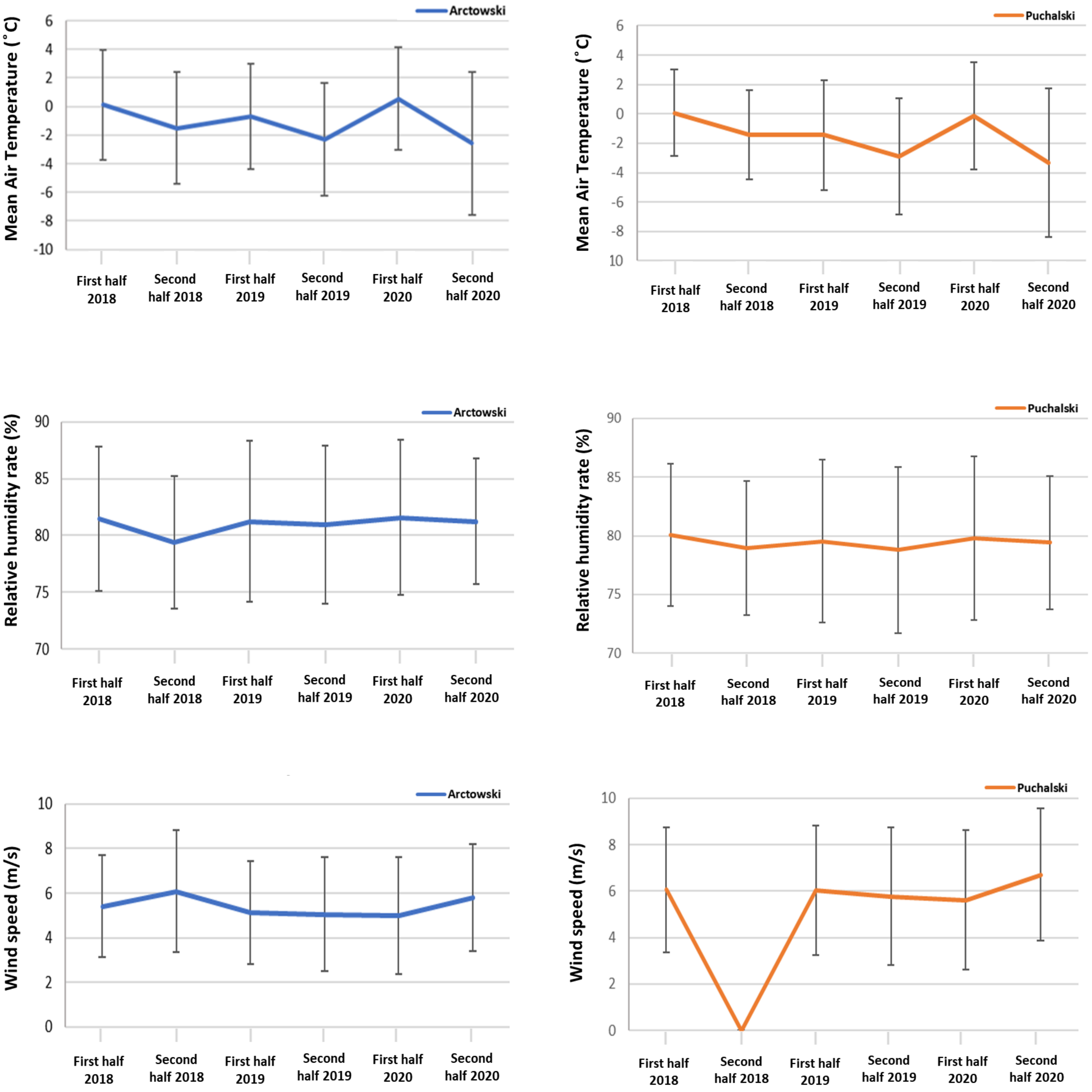

Figure S2. Comparison of climatic data recorded at S1 (Arctowski, blue line) and S2 (Puchalski, orange line) from 2018 to 2020. Wind speed values equal to zero at S2 during the second half of 2018 reflect missing observational data.
