# Supplementary figures and images for: Exploring the molecular landscape of environmental responses in the Antarctic plant Colobanthus quitensis: insights from metatranscriptomic analysis
Source: Front Plant Sci. 2026 Mar 16;17:1774223. doi: 10.3389/fpls.2026.1774223 (PMC13034057; doi:10.3389/fpls.2026.1774223)

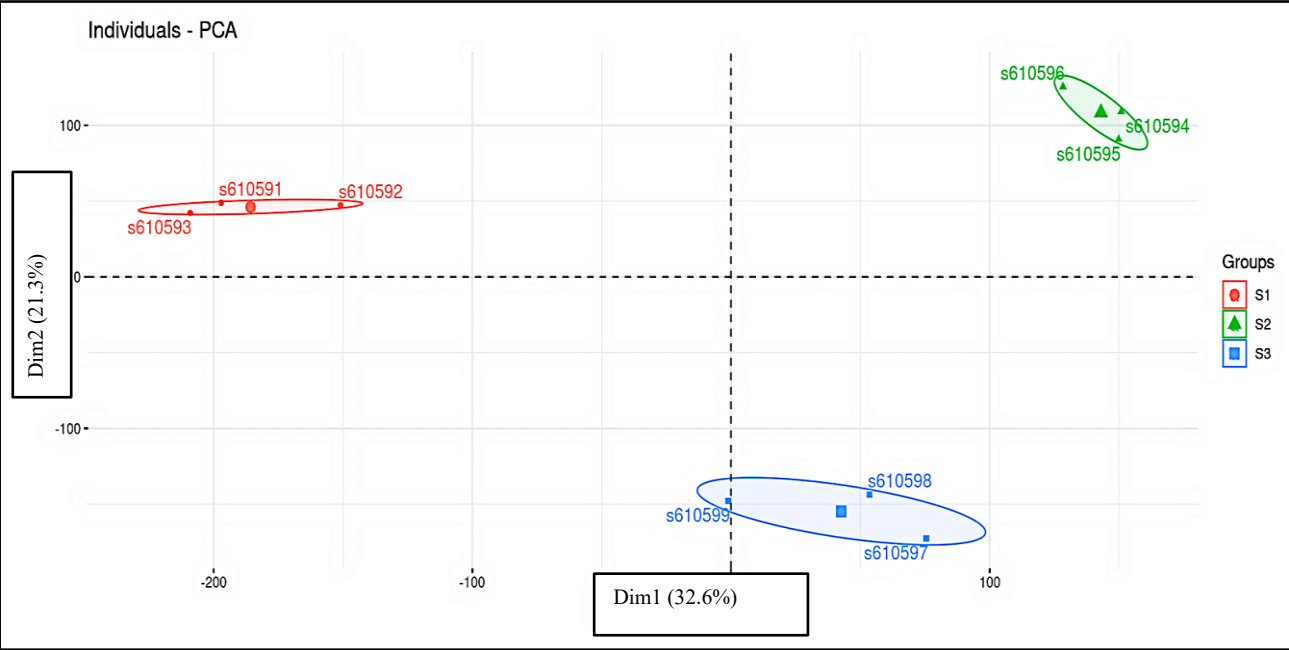

**Figure S3.** PCA analysis

Supplement: Supplementary file 3 [file Image3.pdf]

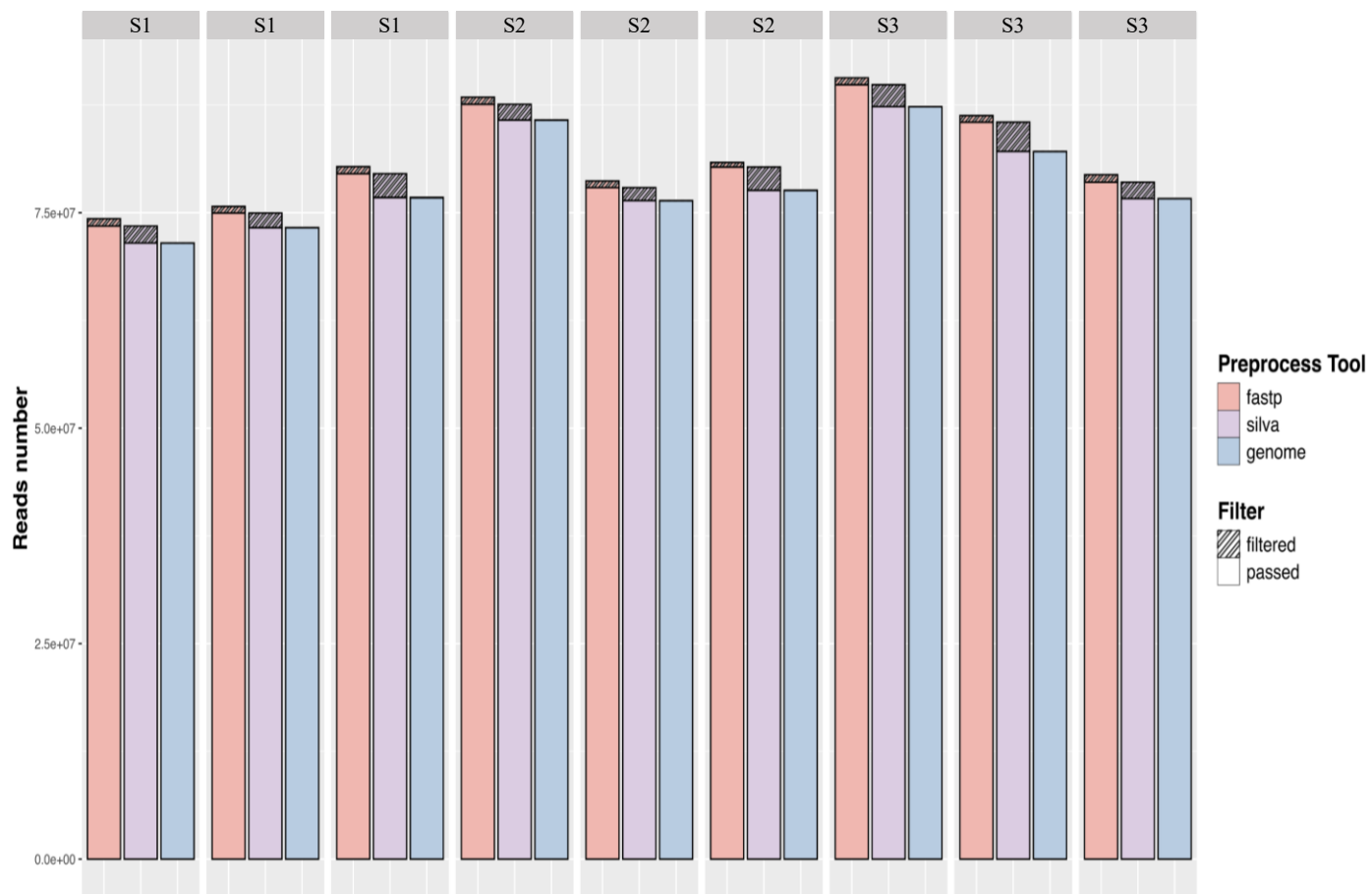

**Figure S4.** Reads distribution in the three sampling sites.

Supplement: Supplementary file 4 [file Image4.pdf]

**A**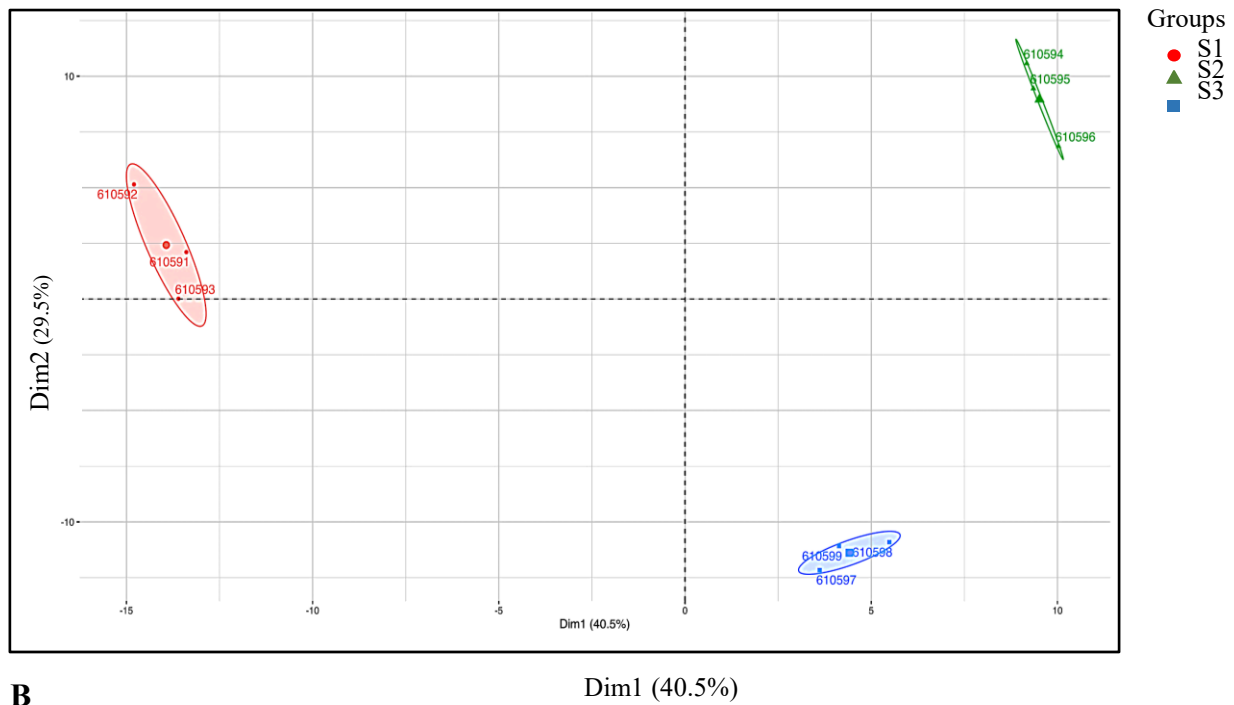**B**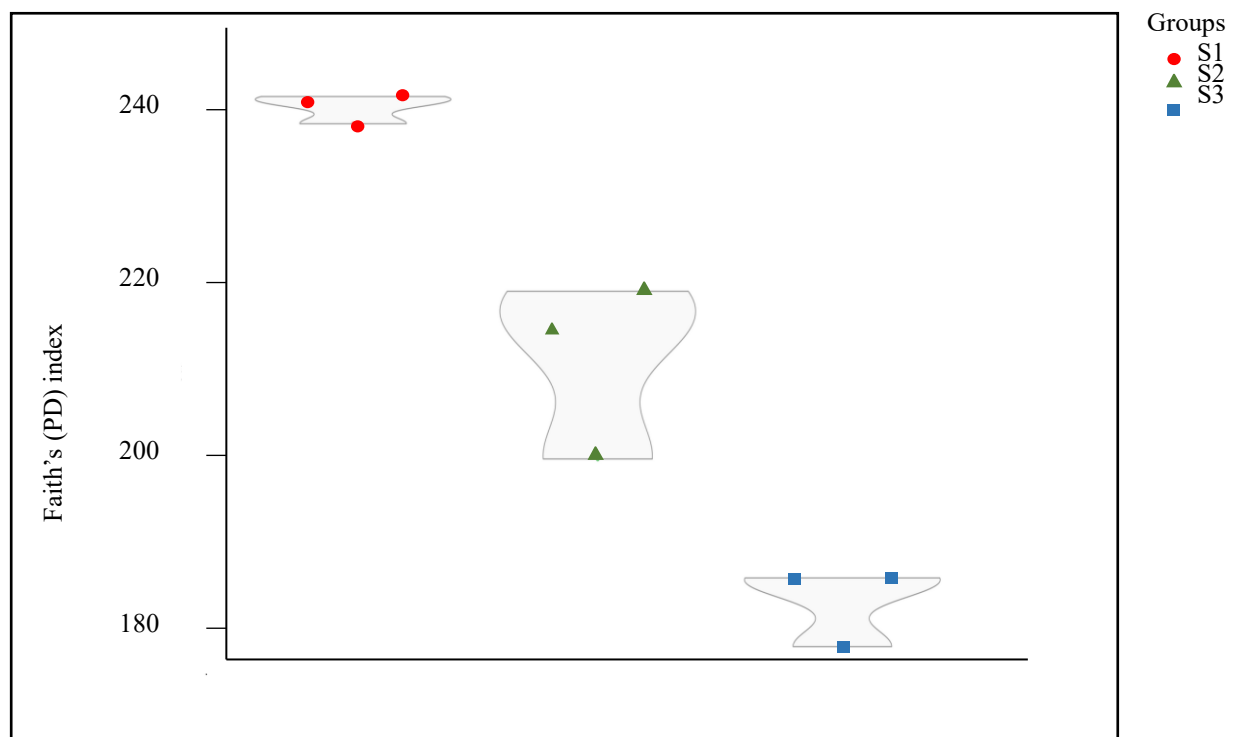

**Figure S5.** PCA analysis (A); Alpha diversity analysis (B).

Supplement: Supplementary file 5 [file Image5.pdf]

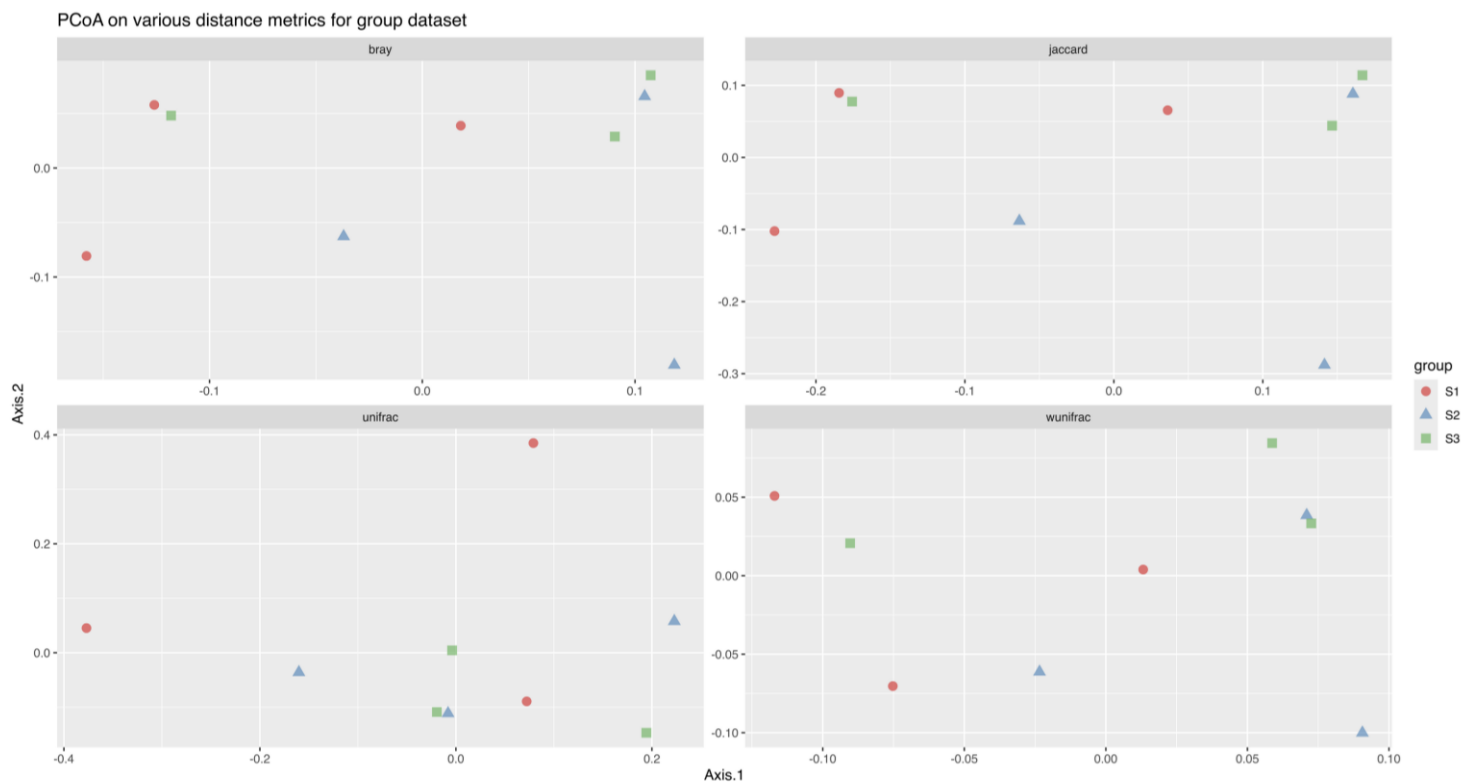

**Figure S6.** Beta diversity analysis.

Supplement: Supplementary file 6 [file Image6.pdf]
